# Geometrically nonlinear high-fidelity aerostructural optimization for highly flexible wings

**DOI:** 10.1007/s00158-025-04181-x

**Published:** 2025-12-09

**Authors:** Alasdair C. Gray, Graeme J. Kennedy, Joaquim R. R. A. Martins

**Affiliations:** 1 Department of Aerospace Engineering, University of Michigan, Ann Arbor, MI USA; 2 School of Aerospace Engineering, Georgia Institute of Technology, Atlanta, GA USA

**Keywords:** Multidisciplinary design optimization, Aeroelasticity, Geometric nonlinearity, High aspect-ratio wing design

## Abstract

Over the past decade, advances in MDO have enabled the aerodynamic and structural design of aircraft wings to be simultaneously optimized using high-fidelity models. Using RANS CFD and detailed structural finite element models in these optimizations enables an accurate trade-off between cruise drag and structural mass. Modeling the coupling of aerodynamics and structures allows the optimizer to aeroelastically tailor the wing, taking advantage of flexibility for improved performance. These capabilities make MDO a key enabling technology for the next generation of flexible and efficient high-aspect-ratio transport aircraft. However, as their aspect ratios increase, these wings increasingly exhibit geometrically nonlinear behavior that linear structural analysis methods cannot model. This work demonstrates the first simultaneous optimization of a wing’s aerodynamic shape and structural sizing using high-fidelity geometrically nonlinear models. To enable this we implement a novel geometrically nonlinear shell element, an efficient nonlinear solver, and a constitutive model for stiffened shells. We then couple these nonlinear structural analysis tools to CFD through a geometrically nonlinear transfer scheme. Using these capabilities, we optimize a single-aisle commercial transport aircraft wing with 547 design variables and 1277 constraints. Although the optimized designs exhibit extreme flexibility—an aspect ratio above 19 and deflections exceeding 30% semispan—geometric nonlinearity has minimal impact on aerodynamic performance, planform design, and overall aircraft mass. However, the Brazier effect causes internal loads that linear analysis misses, requiring geometrically nonlinear analysis to produce a feasible design. The developed framework enables the pursuit of next-generation high-aspect-ratio wing designs by providing the computational foundation needed to exploit extreme wing flexibility as a design opportunity rather than a constraint.

## Introduction

**Fig. 1:**
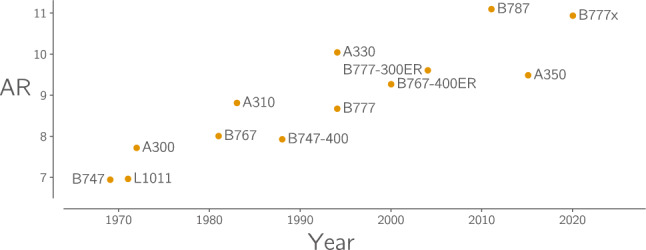
The aspect ratio of commercial transport aircraft wings has increased 50% in the past 50 years [data from Castellani et al. (2017)]

Aircraft manufacturers strive to design and build more efficient aircraft. One of the simplest ways to increase aircraft aerodynamic efficiency is to increase wing aspect ratio. Consequently, the past five decades have seen a steady increase in the aspect ratio of commercial aircraft wings, as shown in Fig. 1.

Key to these increases has been the development of novel airframe technologies, such as advanced composite materials, passive load alleviation through aeroelastic tailoring of wing structures, and active load alleviation through control laws. The use of these technologies in modern airframes has itself been enabled by advances in the computational tools used to design them. As the aspect ratio of a wing increases, it becomes more flexible. This leads to stronger coupling between the structural and aerodynamic behavior, making coupled aerostructural analysis more important. The increasing accessibility of tools, such as NASTRAN, early in the design cycle has enabled the aircraft industry to transition from viewing this aeroelastic coupling as a problem to be avoided, to an opportunity to be exploited.

Following in the wake of these increasingly accessible computational tools has been the application of multidisciplinary design optimization (MDO) to aeroelastic and aerostructural design.^1^ MDO enables more efficient wing designs by optimizing aerodynamic shape and structural sizing simultaneously while considering their coupled effects, rather than using sequential design processes (Chittick and Martins 2009). This approach proves essential for flexible high-aspect-ratio wing (HARW) due to their tight aerodynamic-structural coupling and the increased design parameters introduced by their enabling technologies. As Shirk et al. (1986) noted, “although optimization techniques are very useful in metal design problems, they are almost essential for the efficient design of composite structures.”

Due to the replacement of engineering intuition with simulation results, the computational methods used in MDO frameworks must be capable of modeling the physical phenomena that constrain the design space in reality. Otherwise, optimizers tend to take advantage of gaps in the modeled physics, producing unrealistic optimal designs. In aerostructural optimization of HARW, this leads to two challenges:

1. Most aerostructural optimization problems involve trading off structural weight, peak stress levels, and cruise drag, thus requiring models that can accurately predict these quantities. These accurate predictions can only be achieved using detailed structural finite element (FE) models coupled to aerodynamic models that capture viscous and compressible flow effects, such as Reynolds-averaged Navier–Stokes (RANS) computational fluid dynamics (CFD).
2. HARW exhibit geometrically nonlinear behavior, due to their high flexibility, that cannot be correctly modeled using linear FE methods.

This work demonstrates the first aerostructural optimization using RANS CFD and geometrically nonlinear built-up structural models, addressing both challenges simultaneously. In doing so, we advance toward the grand challenge defined by Slotnick et al. (2014) in the CFD 2030 technology development plan, to enable “multidisciplinary design analysis and optimization (MDAO) of a highly flexible advanced aircraft configuration” using CFD-based approaches.

### Background

Linear structural analysis methods rely on the assumption that the displacements, strains, stresses, and forces in a structure are linearly related. For many engineering applications, where structures should remain in the linear elastic regime and undergo small displacements, this is a good assumption and simplifies any analysis to the solution of a single system of linear equations that describes the equilibrium of external and internal forces on the structure *in its undeformed configuration*. However, under large deformations, and particularly large rotations, these assumptions break down, and geometrically nonlinear analysis is required, in which internal and external forces are balanced *in the structure’s deformed configuration*. Wingboxes, which are composed of thin shell structures, exhibit strong geometric nonlinearity under large deformations through three primary mechanisms:

*Large rotations*: Structural elements that have a large discrepancy between in-plane and out-of-plane stiffness, such as shells and beams, are sensitive to large out-of-plane rotations that significantly reorient the structure’s predominantly stiffened axes.
*Stress stiffening*: Like a taught cable, in-plane stresses are coupled with out-of-plane stiffness. Tensile stresses increase stiffness, while compressive stresses decrease stiffness, potentially leading to buckling, a critical failure mode for shell structures.
*Follower forces*: Wings are primarily subject to aerodynamic pressure loads, which remain normal to the wing’s surfaces as they deform and rotate.

**Fig. 2:**
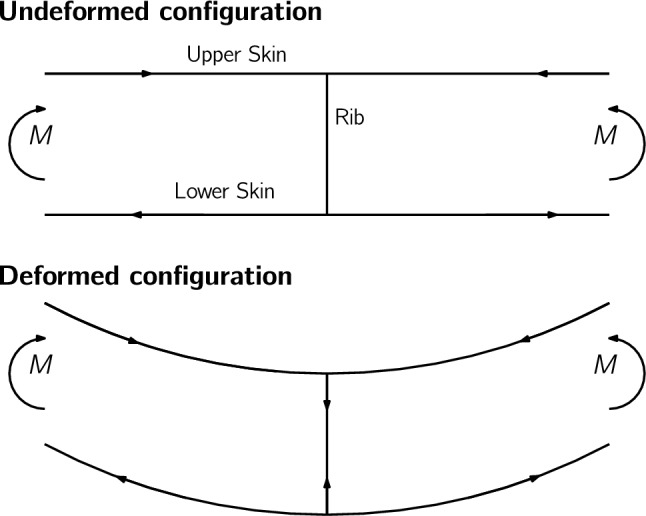
When the curvature of a wingbox under bending is accounted for, the compressive and tensile stresses in the upper and lower skins produce a crushing load in the ribs

Bending in thin-walled beams creates curvature that reorients tensile and compressive stresses in the cross-section, producing crushing pressure that flattens the cross-section. This effect was discovered by engineers building early aircraft wings from thin steel shells and is named after Brazier (1927) who first published work on phenomena and showed how it can cause cross-section buckling. As shown in Fig. 2, the Brazier effect is only captured if the deformed configuration of the beam is considered, and is well known to cause large compressive loads in a wing’s ribs (Gray and Martins 2021; Verri et al. 2025). Structural optimization of wingboxes using linear FE models therefore produces undersized rib designs (Stanford and Dunning 2015; Gray and Martins 2021).

When aeroelastic effects are included, linear structural models may lead to inaccurate sizing not only due to this direct inaccuracy in the linear stress calculation but also to inaccuracy in the deflected wing shape, which affects the aerodynamic load distribution over the wing. Mixing geometrically linear and nonlinear aerodynamic and structural models can lead to significant errors. For example, correctly modeling aerodynamic forces as follower forces results in larger deformations with a nonlinear structural model but lower deformations with a linear structural model (Howcroft et al. 2016). Using a linear structural model with an aerodynamic model that computes forces in the deformed configuration can cause an overprediction of lift because the reduction in the effective span that occurs under large bending deformations is not captured. Finally, while in linear aeroelasticity, drag forces are of little influence, under large bending deformations, drag forces on the outboard portion of the wing induce torsion due to the vertical offset between the wing root and tip. This coupling of chordwise bending and torsion can cause significant changes in static trim (Garcia 2005) and flutter onset (Gray et al. 2023).

Table 1 summarizes work in the past two decades that has pushed the state-of-the-art in aeroelastic analysis and optimization through the inclusion of higher fidelity or geometrically nonlinear models.

**Table 1:** Summary of the literature on high-fidelity and geometrically nonlinear aeroelasticity

Author/Tool	Aerodynamic model^a,b^	Structural model ^b,c^	Optimization variables		
			Structure	Planform	Shape
Smith et al. (2001)	***RANS***	***NL Shell***			
Garcia (2005)	***RANS***	***NL Shell***			
Bartels et al. (2015)	***RANS***	***NL Shell***			
Stickan et al. (2018)	***RANS***	***NL Shell***			
Zimmer et al. (2022)	***RANS***	***NL Shell***			
Verri et al. (2020, 2025)	***RANS***	***NL Shell***			
Calderon et al. (2018, 2019)	IPM	NL Beam	$\checkmark$		
PROTEUS ^d^	IPM	NL Beam	$\checkmark$		
Jovanov (2019)	*Euler*	NL Beam	$\checkmark$		
SU2 (Gomes and Palacios 2022)	***RANS***	***NL Solid***	$\checkmark$		
UM/NAST (Lupp and Cesnik 2019)	ST	NL Beam	$\checkmark$	$\checkmark$	
Conlan-Smith and Andreasen (2022)	IPM	NL Beam	$\checkmark$	$\checkmark$	$\checkmark$
FEMWET (Ma et al. 2022, 2023)	*Q3D*	NL Beam	$\checkmark$	$\checkmark$	$\checkmark$
Stanford et al. ^e^	IPM	***Shell***	$\checkmark$		
Stanford (2020)	IPM	***Shell***	$\checkmark$	$\checkmark$	$\checkmark$
Jacobson and Stanford (2022)	***RANS***	***Shell***	$\checkmark$	$\checkmark$	
DLR ^f^	***RANS***	***Shell***	$\checkmark$	$\checkmark$	$\checkmark$
MACH ^g^	***RANS***	***Shell***	$\checkmark$	$\checkmark$	$\checkmark$
Gray and Martins (2021)	***RANS***	***NL Shell***	$\checkmark$		
This work	***RANS***	***NL Shell***	$\checkmark$	$\checkmark$	$\checkmark$

^a^ Aerodynamic models: IPM = Inviscid panel method, ST = Strip theory, Q3D = Quasi-3D, Euler = Euler CFD, RANS = RANS CFD

^b^ Fidelity: Low, Mid, ***High***

^c^ Structural models: NL = Nonlinear

^d^ (Werter and De Breuker 2016; Krüger et al. 2019; Rajpal et al. 2019; Bordogna et al. 2020)

^e^ (Stanford and Dunning 2014, 2015; Stanford et al. 2015; Stanford 2017, 2018; Stanford et al. 2018)

^f^ (Abu-Zurayk et al. 2020; Wunderlich et al. 2021, 2022)

^g^ (Kenway et al. 2014b; Kenway and Martins 2015; Burdette and Martins 2019; Brooks et al. 2020)

Several authors have developed tools capable of gradient-based aeroelastic optimization using geometrically nonlinear beam models coupled to low-fidelity aerodynamic models such as strip theory or inviscid panel models. Examples include the PROTEUS framework, developed by Werter and De Breuker (2016), and UM/NAST, developed by Su and Cesnik (2010), The low cost of these computational models allows for the inclusion of constraints based on complex analyses that are typically too costly to include with higher-fidelity models, such as flutter stability (Lupp and Cesnik 2019; Rosatelli et al. 2024), gust loads (Rajpal et al. 2019) and fatigue-life constraints (Rajpal et al. 2019). However, their use of low-fidelity methods limits their ability to accurately trade-off aerodynamic drag and structural mass, unless higher-fidelity models are also included in the optimization process (Gray et al. 2023).

Gomes and Palacios (2022) demonstrated the capability to perform topology optimization of the internal structure of a solid wing using a geometrically and materially nonlinear continuum FE model coupled to RANS CFD using SU2. However, as the authors acknowledge, their tool’s ability to design practical wings is limited as they can only optimize the structure considering a single flight point. Additionally, solid continuum elements are not suitable for modeling realistic aircraft structures made from thin shells at a practical computational cost.

Ma et al. (2022, 2023) extended the FEMWET code developed by Elham and van Tooren (2016) to include a geometrically nonlinear composite beam model and applied it to the design of strut-braced-wing and twin-fuselage aircraft. FEMWET couples a geometrically nonlinear beam model to a quasi-3D aerodynamic model that uses a vortex lattice method (VLM) for lift and induced drag prediction, and 2D compressible, viscous analysis of the wing’s cross sections to predict viscous and wave drag. The inclusion of viscous and compressible aerodynamics in FEMWET allows for meaningful aerostructural optimization of the wing’s structure, planform, and section shapes. While this is an improvement over the other low-fidelity tools previously discussed, these models are still less accurate than RANS CFD and built-up structural FE models. They are also limited to modeling isolated wings, while higher-fidelity tools can more easily model complete aircraft configurations.

Although these tools have been used to optimize wing structures based on geometrically nonlinear aeroelastic analysis, few works have explicitly investigated the differences in wing designs optimized with and without considering such effects. Calderon et al. (2018, 2019) studied these implications using the NeoCASS framework (Cavagna et al. 2011), which couples geometrically nonlinear beams with a VLM. Their iterative sizing process is not a fully coupled aeroelastic or aerostructural optimization, but is more similar to the processes used in industry. In each iteration, the wing’s twist distribution is chosen to achieve a pre-defined cruise lift distribution, aeroelastic maneuver analyses are then performed, and the structure is sized based on the resulting (fixed) loads. They repeated this process using both linear and geometrically nonlinear structural models across various wing aspect ratios. They found that wings sized using geometrically nonlinear structural analysis were lighter over the entire range of aspect ratios studied, had roughly equal aerodynamic efficiency, and had a higher optimum aspect ratio than those sized using linear structural analysis.

Smith et al. (2001) and Garcia (2005) both demonstrated the importance of the drag-torsion coupling discussed earlier using nonlinear beam models and RANS CFD. Today, coupling RANS CFD with nonlinear shell FE models is the state-of-the-art in high-fidelity geometrically nonlinear aeroelastic modeling of realistic aircraft configurations. Stickan et al. (2018), Zimmer et al. (2022), and Verri et al. (2020, 2024) demonstrated this capability, by coupling TAU and Abaqus at Airbus, TAU and NASTRAN at DLR, and CFD++ and NASTRAN at Embraer, respectively. These works showed that geometrically nonlinear effects are visible in wings with aspect ratios typical of the latest generation of commercial transport aircraft.

The current state-of-the-art in high-fidelity aerostructural optimization involves coupled RANS CFD and linear shell FE models, though only a handful of tools are capable of this. The MACH (MDO of aircraft configurations at high-fidelity) framework is one such tool (Kenway et al. 2014b). MACH’s coupled adjoint gradient computation implementation has enabled the solution of large-scale aerostructural optimization problems with $\mathcal {O}\left( 10^3\right)$ design variables parametrizing the wing structure and geometry and $\mathcal {O}\left( 10^3\right)$ constraints involving analyses at $\mathcal {O}\left( 10\right)$ flight conditions. The framework has been used to investigate the potential benefits of several HARW-enabling airframe technologies such as wing morphing (Burdette and Martins 2018b, 2019) and tow-steered composites (Brooks and Martins 2018a; Brooks et al. 2020). Various groups at DLR have demonstrated similar capabilities. Wunderlich et al. (2021, 2022) performed aerostructural optimization of the Airbus XRF1 configuration including maneuver load alleviation and landing gear integration. Their process involves nested optimization, where an outer optimizer controls the aircraft geometry, and an inner optimization process sizes the wing structure using the popular commercial tools NASTRAN and Hypersizer. The disadvantages of this approach are that the number of geometric design variables is limited because the outer optimizer is gradient-free, and that the process is not guaranteed to converge to the true aerostructural optimum because the structure is optimized under the assumption of fixed loads. Abu-Zurayk et al. (2020) use a monolithic MDO architecture similar to MACH but that also includes a propulsion model coupled to the CFD and a comprehensive set of structural loads generated using lower fidelity tools. Finally, Jacobson and Stanford (2022) performed aerostructural optimization including flutter constraints computed using Linearized frequency domain (LFD) CFD. The scale of their optimization problems was limited by the high cost of computing gradients through the LFD analysis.

There is therefore a gap in state-of-the-art aerostructural optimization tools: low-fidelity tools include geometric nonlinearities while high-fidelity tools do not. In previous work (Gray and Martins 2021), we attempted to bridge this gap by incorporating geometrically nonlinear shell elements into the MACH framework, enabling geometrically nonlinear coupled analysis and optimization using RANS CFD and shell FE models.

Using this framework, we demonstrated the first detailed wingbox sizing optimization using coupled high-fidelity RANS CFD and geometrically nonlinear shell FE models. However, this work was subject to two limitations:

1. The load and displacement transfer scheme was not geometrically nonlinear.
2. Derivatives with respect to node coordinates were not available for the nonlinear shell elements, meaning only the wing’s structural sizing was optimized, while the geometry remained fixed.

In this work we eliminate these shortcomings and demonstrate the first framework for high-fidelity aerostructural optimization that is fully geometrically nonlinear and includes both structural and geometric design variables. We implement a novel nonlinear shell element formulation (Sect. 2.1), nonlinear solver (Sect. 2.2), and stiffened shell constitutive model (Sects. 2.3 and 2.4) for robust and efficient geometrically nonlinear analysis and optimization in the open-source FE library TACS. We describe how TACS is coupled to the RANS CFD solver ADflow using a geometrically nonlinear load and displacement transfer scheme (Sect. 3.2), and how we efficiently solve the resulting coupled primal and adjoint equations (Sect. 3.4). We use a benchmark single-aisle transport aircraft wing design problem (Sect. 4) to demonstrate the capabilities of our tools and discuss the implications of geometrically nonlinear analysis on aerostructural optimization (Sect. 5).

## Geometrically nonlinear structural analysis

For structural analysis, we use TACS^2^ (Kennedy and Martins 2014), an efficient parallel FE library that is particularly effective at solving the poorly conditioned systems of equations that result from the thin-walled structures typical of airframes. TACS uses a fully analytic adjoint implementation to compute derivatives of functions of interest, such as aggregated stress constraints, efficiently with respect to large numbers of design variables. The version of TACS used in our previous work with MACH was an older version of the code that featured a different nonlinear element implementation that could not compute geometric derivatives. In this work, we use the latest open-source version of the code.

### Nonlinear shell elements

The nonlinear shell elements in TACS use a mixed interpolation of tensorial components (MITC) formulation for the out-of-plane shear and in-plane normal strain components that are susceptible to shear or membrane locking (Dvorkin and Bathe 1984; Bathe and Dvorkin 1986). The shell elements implement a nonlinear drilling penalization term that is designed to ensure consistency between the in-plane rotation and rotation about the shell normal (Simo et al. 1992; Fox and Simo 1992). The formulation uses all linear and nonlinear contributions to the green strain for the in-plane, bending, and shear strain components. A director field formulation parameterizes the rate-of-change of the displacements through the thickness of the shell (Simo and Fox 1989; Simo et al. 1990). We compute this director field using a quadratic approximation of a rotation matrix applied to the shell unit normal, where the matrix is computed using the degrees of freedom at each node. Given the rotational degrees of freedom $\theta \in \mathbb {R}^{3}$, we compute the quadratic approximation of the rotation matrix $\boldsymbol{C}$ using the skew-symmetric cross-product matrix $\theta ^{\times }$:

1 $$\begin{aligned} \begin{aligned} \boldsymbol{C}(\theta )&= \boldsymbol{I} + \theta ^{\times } + \frac{1}{2} \theta ^{\times } \theta ^{\times } \\&= (1 - \frac{1}{2} \theta ^{T}\theta ) \boldsymbol{I} + \theta ^{\times } + \frac{1}{2} \theta \theta ^{T} \\&\approx \boldsymbol{C}_{\text {exact}}(\theta ) = \exp (\theta ^{\times }) = \sum _{k=0}^{\infty } \frac{1}{k!} \left( \theta ^{\times } \right) ^{k} \end{aligned} \end{aligned}$$

This quadratic approximation of the exponential map enables the modeling of near extension-free moderate rotations. This capability is important for HARWs that experience large tip deflections and moderate rotations. We compute the rate-of-change of displacement through the shell thickness from the rotation (1) as

2 $$\begin{aligned} \boldsymbol{d} = (\boldsymbol{C}(\theta ) - \boldsymbol{I}) \hat{\boldsymbol{n}} , \end{aligned}$$

where $\hat{\boldsymbol{n}}$ is the shell surface normal and $\boldsymbol{d}$ is the rate-of-change of deformation through the thickness. We interpolate the in-plane displacements and director field (2) across the element from the nodes using standard Lagrange interpolation and use them in combination with the MITC formulation to compute the in-plane, bending and shear strains.

**Fig. 3:**
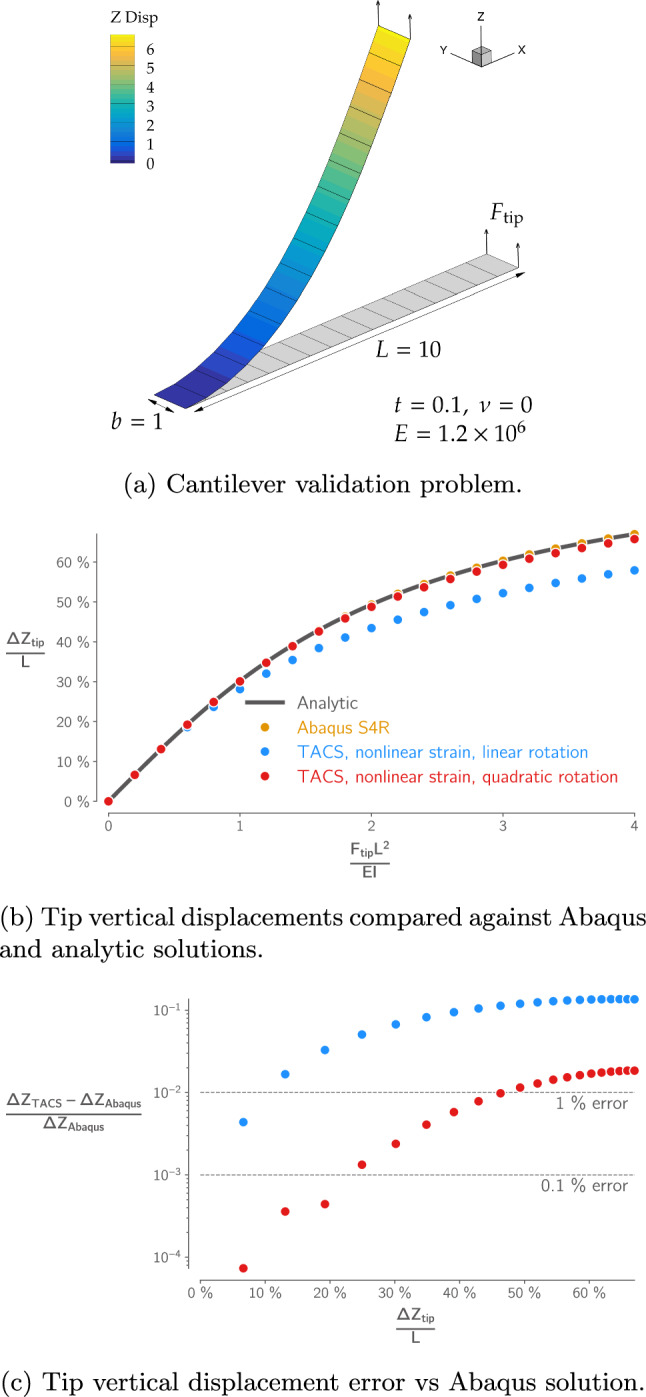
The quadratic rotation approximation used by the TACS shell elements is accurate for moderate deflection levels

To verify the accuracy of the quadratic rotation matrix approximation, we use the popular benchmark problem of a cantilever beam subject to a tip load, shown in Fig. 3a. Figure 3 compares the TACS prediction of the beam’s tip displacement against an analytic solution (Barten 1944) and data computed by Sze et al. (2004) using the commercial FE software Abaqus. The Abaqus data was generated with the same 16 element mesh as the TACS model but using an element formulation (S4R) with an exact rotation treatment. We show results for two formulations of the TACS shell element, one using the quadratic approximation of the rotation matrix and one using a linearized rotation matrix, both using the full nonlinear Green-Lagrange strain.^3^ The formulation using the linearized rotation treatment is only accurate to within 1% of the Abaqus solution^4^ up to a tip deflection of 10% of the beam length, while the quadratic approximation is accurate to the same level up to a tip deflection of almost 50% of the beam length and a tip rotation angle of around 40°. These are both larger than the maximum deflection and rotation seen in the wingboxes we model herein.

### Nonlinear solver

To solve geometrically nonlinear structural problems in TACS we implement a Newton–Raphson based solver, which includes improvements on the solver used in our previous work (Gray and Martins 2021). In nonlinear structural problems we solve for equilibrium in the deformed rather than undeformed configuration, as a result, the equilibrium can no longer be written as a linear system $\boldsymbol{K}\boldsymbol{u}=\boldsymbol{f}$ but should instead be written as a system of nonlinear residual equations,

3 $$\begin{aligned} \boldsymbol{r\left( u\right) } = \boldsymbol{F_\text {in}\left( u\right) } + \boldsymbol{F_\text {ex}\left( u\right) } = 0 . \end{aligned}$$

where $\boldsymbol{u}$ are the nodal displacements, $\boldsymbol{F_\text {in}}$ are the internal forces resulting from stresses, and $\boldsymbol{F_\text {ex}}$ are the externally applied forces. As in our previous work, this solver uses adaptive load incrementation/continuation, meaning the solver is repeatedly called on a modified set of residual equations where the external forces are scaled down by a load factor $\lambda$ as follows:

4 $$\begin{aligned}&\boldsymbol{r}(\boldsymbol{u}, \lambda ) = \boldsymbol{F_\text {in}\left( u\right) } + \lambda \boldsymbol{F_\text {ex}\left( u\right) } = 0 \end{aligned}$$

Using the Newton–Raphson method, we can solve these equations by repeatedly computing a displacement update using the tangent stiffness matrix, $\boldsymbol{K_T}$, a local linearization of the nonlinear residual equations,

5 $$\begin{aligned}&\boldsymbol{K_T}(\boldsymbol{u_i}, \lambda ) = \boldsymbol{\frac{\partial r}{ \partial u}}|_{u=u_i} = \boldsymbol{\frac{\partial F_\text {in}}{\partial u}}\big |_{u=u_i} + \lambda \boldsymbol{\frac{\partial F_\text {ex}}{\partial u}}\big |_{u=u_i} \end{aligned}$$

6 $$\begin{aligned}&\boldsymbol{\Delta u_i} = -\boldsymbol{K_T}(\boldsymbol{u_i}, \lambda ) ^{-1}\boldsymbol{r}(\boldsymbol{u_i}, \lambda ) . \end{aligned}$$

The solution of this system for a given load factor is a point on the equilibrium path of the structure, $\boldsymbol{u^*}(\lambda )$. To control the size of each load increment, we use the adaptive load stepping method of Beluni and Chulya (1987), where the current load step size is increased or decreased based on the actual and desired number of iterations taken to solve the previous increment,

7 $$\begin{aligned} \lambda _j - \lambda _{j-1} = \Delta \lambda _{j} = \sqrt{\frac{N_\text {des}}{N_{j-1}}}\Delta \lambda _{j-1} . \end{aligned}$$

The desired number of iterations, $N_\text {des}$, can then be used to control how ambitious the solver should be with its load incrementation.

Previously, the nonlinear solution for each load increment started from the equilibrium point found in the previous increment, $\boldsymbol{u}_{0,j} = \boldsymbol{u}^*_{j-1}$. In our new implementation, we keep track of the last *n* discovered equilibrium points and use them to extrapolate the next point on the equilibrium path, which is then used as the initial guess for the next Newton–Raphson solution. This reduces the number of iterations required to reach convergence due to the Newton–Raphson method’s sensitivity to the quality of its starting point. Using Lagrange polynomial interpolation, the extrapolated equilibrium point is given by a linear combination of the previous points,

8 $$\begin{aligned} \boldsymbol{u}_{0,j} = \sum _{i=0}^{n-1} \boldsymbol{u}^*_{i} \prod _{k=0, k\ne i}^{n-1} \frac{\lambda _j - \lambda _k}{\lambda _i - \lambda _k} , \end{aligned}$$

which is inexpensive to compute. This approach can be considered a predictor-corrector continuation method, where the equilibrium path extrapolation is the predictor stage and the Newton–Raphson solution the corrector stage.

**Fig. 4:**
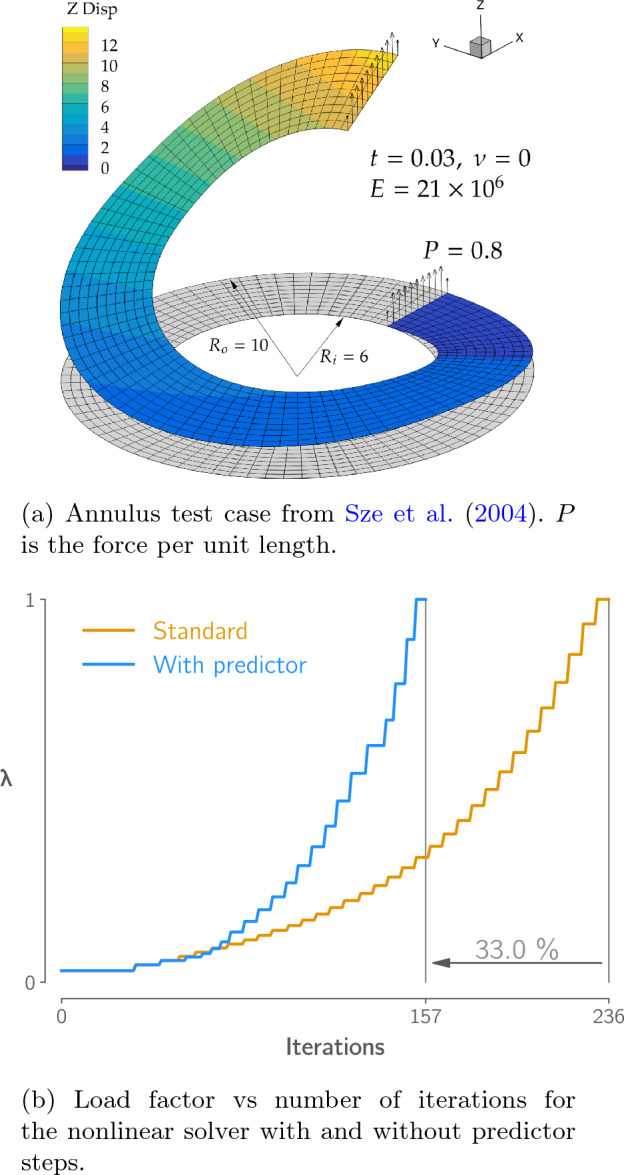
Extrapolating the equilibrium path allows the Newton–Raphson solver to converge larger load increments in the same number of iterations, requiring 33% fewer iterations in this case

To demonstrate the effectiveness of the predictor step, we compare the number of iterations taken to solve another benchmark problem from Sze et al. (2004), involving an annular shell (shown in Fig. 4a), with and without the predictor step.^5^ We use the same 10$\times$80 mesh as Sze et al. (2004). Both solutions start with an initial load step size of $\Delta \lambda = 0.03$ and $N_{des} = 6$. With the predictor steps enabled, we use $n=4$ previous equilibrium points to extrapolate the next point on the equilibrium path. Figure 4b shows that, with the predictor providing a good initial guess, the Newton–Raphson can converge larger load increments in the same number of iterations. Fewer increments are therefore required to reach the final load factor of $\lambda = 1.0$, resulting in a 30% reduction in the number of iterations required to reach the solution. The number of iterations required to reach the solution in both cases is substantially lower than the 346 iterations reported by Sze et al. (2004) for the same problem using Abaqus.

Our second improvement over our previous implementation is the use of adaptive linear convergence. Due to the extremely poor conditioning of shell structural models, TACS solves the linear system in Eq. (6) using GMRES preconditioned by a complete LU factorization. This means that the most expensive operation in the solver is recomputing the linear system preconditioner. Therefore, during each Newton–Raphson solution, we update the tangent stiffness matrix every iteration but only recompute the preconditioner if the previous linear solution took more than a specified number of iterations to converge to the desired tolerance. In combination with this, we adapt this desired tolerance using the variant of the Eisenstat–Walker method (Eisenstat and Walker 1996) proposed by An et al. (2007). This method avoids “over-solving" the linear system early in the Newton–Raphson solution and tightens the convergence tolerance as the nonlinear solver approaches a solution. These two approaches are complementary since it is generally not possible to solve the linear system tightly with an out-of-date preconditioner, and updating the preconditioner every iteration generally results in very tight linear convergence in only 1-2 GMRES iterations.

**Fig. 5:**
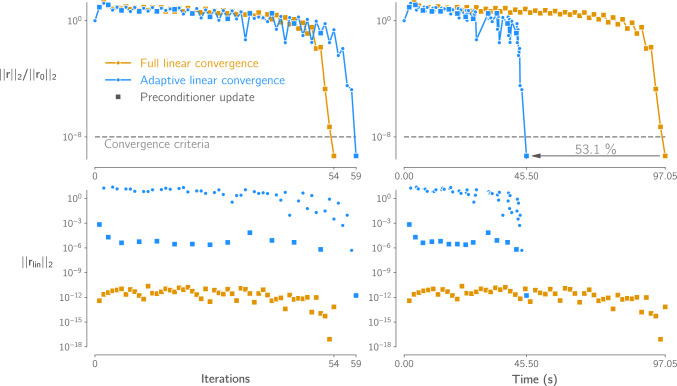
Adaptive linear convergence speeds up the nonlinear solver, in this case by over 50%

Figure 5 demonstrates the effectiveness of this approach on the geometrically nonlinear hemispherical shell benchmark problem described by Sze et al. (2004)^6^.^7^ The plots show the nonlinear (top) and linear (bottom) residual in each Newton–Raphson iteration during the solution. Although using adaptive linear convergence requires more Newton–Raphson iterations to converge, the time to reach the solution is reduced by over 50% due to the reduced number of LU factorizations required.

Finally, we retain the use of the novel minimum energy restart method for our load continuation scheme (Gray and Martins 2021). This method is used to compute the load factor to start at when restarting the continuation from a previous solution by computing the load factor that minimizes the work done in the resulting Newton–Raphson step,

9 $$\begin{aligned} \lambda ^*= & \frac{-\left( \boldsymbol{\Delta u_i}^\text {T}\boldsymbol{F_\text {ex}} + \boldsymbol{\Delta u_e}^\text {T}\boldsymbol{F_\text {in}} \right) }{2\boldsymbol{\Delta u_e}^\text {T}\boldsymbol{F_\text {ex}}},\nonumber \\ & \boldsymbol{\Delta u_i} = \boldsymbol{K_T}^{-1} \boldsymbol{F_\text {in}}, \ \boldsymbol{\Delta u_e} = \boldsymbol{K_T}^{-1} \boldsymbol{F_\text {ex}}. \end{aligned}$$

This technique is particularly useful during optimization, where the same analysis may be repeated 1000 s of times with slightly modified external loads and structural sizing parameters.

### Stiffened shell constitutive model

Practical optimization of aerospace structures requires the ability to model stiffened composite shells. We model these shells using a constitutive model based on first-order shear deformation theory^8^ ,valid for moderately thick shells with non-negligible transverse shear deformations. Rather than modeling stiffeners explicitly, we use a smeared stiffener approach where the stiffness of the stiffener is smeared over the shell following the theory of Nemeth (2011). While this approach does not capture the load concentration that occurs at the stiffeners, it is advantageous for optimization applications because it allows both the stiffener cross section and spacing to be optimized without modifying the structural mesh. It is therefore most suitable for conceptual or early preliminary design stages, where the structural layout is not yet fixed. A similar approach has been used by Kennedy et al. (2014a), Brooks et al. (2019), and Dähne et al. (2024). Dähne and Werthen (2018) verified their implementation of this smeared stiffness approach against explicitly modeled T-shaped stiffeners, confirming its accuracy for stiffness and buckling prediction.

The resulting overall stiffness matrix for the stiffened shell is

10 $$\begin{aligned} \boldsymbol{C} = \begin{bmatrix} \boldsymbol{A_\text {sh}} & \boldsymbol{B_\text {sh}} & \boldsymbol{0} \\ \boldsymbol{B_\text {sh}} & \boldsymbol{D_\text {sh}} & \boldsymbol{0} \\ \boldsymbol{0} & \boldsymbol{0} & \boldsymbol{A_{s,\text {sh}}} \end{bmatrix} + \frac{1}{P_\text {st}} \begin{bmatrix} \boldsymbol{A_\text {st}} & \boldsymbol{B_\text {st}} & \boldsymbol{0} \\ \boldsymbol{B_\text {st}} & \boldsymbol{D_\text {st}} & \boldsymbol{0} \\ \boldsymbol{0} & \boldsymbol{0} & \boldsymbol{A_{s,\text {st}}} \end{bmatrix}, \end{aligned}$$

where

11 $$\begin{aligned} \boldsymbol{A_\text {st}}&= \begin{bmatrix} A_\text {st}E_\text {st} & 0 & 0 \\ 0 & 0 & 0 \\ 0 & 0 & \frac{kG_\text {st}A_\text {st}}{4} \end{bmatrix} \end{aligned}$$

12 $$\begin{aligned} \boldsymbol{B_\text {st}}&= \begin{bmatrix} A_\text {st}E_\text {st}Z_c & 0 & 0 \\ 0 & 0 & 0 \\ 0 & 0 & \frac{kG_\text {st}A_\text {st}Z_c}{4} \end{bmatrix} \end{aligned}$$

13 $$\begin{aligned} \boldsymbol{D_\text {st}}&= \begin{bmatrix} E_\text {st}(I_{22,\text {st}} + A_\text {st} Z_c^2) & 0 & 0 \\ 0 & 0 & 0 \\ 0 & 0 & \frac{G_\text {st}(J_\text {st} + kA_\text {st}Z_c^2)}{4} \end{bmatrix} \end{aligned}$$

14 $$\begin{aligned} \boldsymbol{A_{s,\text {st}}}&= \begin{bmatrix} 0 & 0 \\ 0 & kG_\text {st}A_\text {st} \end{bmatrix}. \end{aligned}$$

Here, $A_\text {st}$ is the area, $I_{33,\text {st}}$ the second moment of area and $J_\text {st}$ the polar moment of inertia of the stiffener cross-section about its centroid. *k* is the shear correction factor. This formulation is similar to that used in TACS previously (Kennedy et al. 2014a; Brooks et al. 2019), except for the additional $B_{66}$ and $D_{66}$ terms. Further details on the derivation of this formulation are given in Appendix 1.

**Fig. 6:**
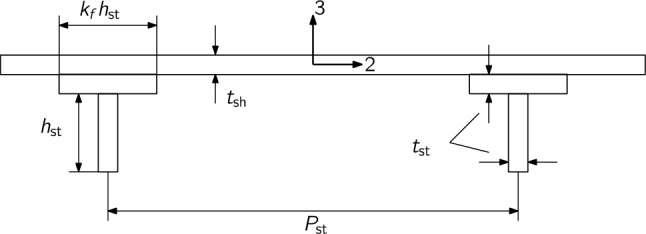
Stiffened panel cross-section

In this implementation we assume a T-shaped stiffener cross-section as shown in Fig. 6, and compute $A_\text {st}, I_{22,\text {st}}, J_\text {st}, Z_c$ accordingly. The implementation allows the following design variables for optimization:

- Laminate thicknesses of the panel, $t_\text {sh}$.
- Stiffener web and flange thicknesses, $t_\text {st}$.
- Ply fractions of the panel and stiffeners laminates, $f_i$.
- Stiffener height, $$h_\text {st}$$.
- Stiffener pitch, $$P_\text {st}$$.

We compute the necessary derivatives of the panel stresses and stiffness with respect to these design variables analytically.

### Failure criteria

In our stiffened panel model, we consider several different failure modes, covering both material and buckling failure in the skin and stiffeners. All of our failure criteria are framed in terms of a strength ratio, *SR*, which is the inverse of the safety factor.^9^ All of these strength ratios are combined using KS aggregation (Kreisselmeier and Steinhauser 1979) to produce a single value for each integration point in the FE mesh.

To predict material failure, we use the Tsai–Wu criterion^10^ (Tsai and Wu 1971), which predicts failure when

15 $$\begin{aligned} & F_1\sigma _{11} + F_2\sigma _{22} + F_{11}\sigma _{11}^2 + F_{22}\sigma _{22}^2 \nonumber \\ & \quad + F_{66}\sigma _{12}^2 + F_{12}\sigma _{11}\sigma _{22} = \boldsymbol{\boldsymbol{\sigma }}^\text {T} \boldsymbol{F} \boldsymbol{\boldsymbol{\sigma }} + \boldsymbol{f}^\text {T} \boldsymbol{\boldsymbol{\sigma }}=1. \end{aligned}$$

For a given stress state, we compute the Tsai–Wu strength ratio by introducing a scaling factor of 1/*SR* on the stresses and solving for the value of *SR* that satisfies Eq. (15), yielding

16 $$\begin{aligned} SR = 1/2 \left( \boldsymbol{f}^\text {T} \boldsymbol{\boldsymbol{\sigma }} + \sqrt{(\boldsymbol{f}^\text {T} \boldsymbol{\boldsymbol{\sigma }})^2 + 4\boldsymbol{\boldsymbol{\sigma }}^T \boldsymbol{F} \boldsymbol{\boldsymbol{\sigma }}}\right) . \end{aligned}$$

At each integration point in the FE mesh, we compute this strength ratio at the top and bottom surfaces of the skin, and the top and bottom of the stiffener. Since we assume that the plies of each angle are distributed evenly through the thickness, we repeat these failure calculations for all ply angles.

Despite being one of the simpler proposed theories, the Tsai–Wu criterion has proven to be at least as accurate as more complex theories for many cases of combined in-plane loading on unidirectional laminae (Soden et al. 2004). These plane stress conditions are typical of the walls of thin shell structures, such as the stiffened panels we model in this work. When laminates are subject to 3D stress states, or when detailed failure progression must be simulated, more complex analyses use separate criteria for different failure modes, such as the Puck (Deuschle and Puck 2013) or LaRC methods (Pinho et al. 2012). However, these analyses require more extensive experimental data and are incompatible with gradient-based optimization (Hermansen and Lund 2024).

Our model can also use a simpler maximum strain criterion for laminate failure. This criterion is often used in industrial preliminary structural optimization because the strain allowables are simpler to modify to account for more complex constraints such as required damage tolerance (Iorga et al. 2016).

We also compute two strength ratios related to panel buckling failure, one for skin buckling between stiffeners and one for global panel buckling. Both buckling strength ratios combine axial and shear buckling loads into a single value,

17 $$\begin{aligned} SR= & 1/2 \left( \frac{N_1}{N_{1,\text {crit}}} \right. \nonumber \\ & \quad + \left. \sqrt{\left( \frac{N_1}{N_{1,\text {crit}}}\right) ^2 + 4\left( \frac{N_{12}}{N_{12,\text {crit}}}\right) ^2}\right) \end{aligned}$$

where $N_1$ and $N_{12}$ are the axial and shear loads on the panel, and $N_{1,\text {crit}}$ and $N_{12,\text {crit}}$ are the critical axial and shear loads for buckling. These critical loads for global and local panel buckling are computed using equations given by Stroud and Agranoff (1976).^11^

Finally, we include two potential isolated failure modes for the stiffeners. We compute a strength ratio for stiffener column buckling using the Euler critical load for pinned-pinned columns

18 $$\begin{aligned} SR_\text {column} = \frac{F_\text {column}}{F_\text {ult}} = \frac{ A_\text {stiff} \epsilon _{11,\text {stiff}} L^2}{\pi ^2 I_{33,\text {stiff}}}. \end{aligned}$$

For stiffener crippling, we use a semi-empirical formula for one-edge-free crippling (Department of Defense 2002, Chapter 5)

19 $$\begin{aligned} \frac{F_\text {crippling}}{F_\text {ult}} = 1.63\left( \frac{b}{t}\right) ^{-0.717}, \end{aligned}$$

where $F_\text {crippling}$ is the crippling load, $F_\text {ult}$ is the laminate’s ultimate compressive strength, *b* is the flange width or height, and *t* is the flange thickness. We compute the laminate’s ultimate compressive strength using the Tsai-Wu strength ratio described above using only the compressive axial component of the strain. The crippling strength ratio is then

20 $$\begin{aligned} SR_\text {crippling} = \frac{SR_\text {TW}}{1.63}\left( \frac{b}{t}\right) ^{0.717}. \end{aligned}$$

We compute this crippling strength ratio for both the stiffener’s flange and web. When computing the crippling strength ratio for the web, we use the method described by Kassapoglou (2013, section 8.5.3) to account for the variation in stress along the web due to stiffener bending. Specifically, *b* is considered to be the length of only the web section that is in compression, and the strain used to compute $SR_\text {TW}$ is the average axial strain over this section.

As with the stress and stiffness calculations, we compute the derivatives of all the buckling and failure criteria with respect to the shell strains and design variables analytically.

There are several limitations to the failure criteria used in this implementation. The equations used for the critical global panel buckling loads are meant for a flat panel that is infinitely wide in the $x_2$ direction. They therefore do not account for the curvature of the panel (e.g., in a wing skin), nor the stiffening effect of the adjacent panels and are thus conservative. The critical inter-stiffener buckling loads are also conservative, as they ignore the stiffening effect of the stiffener flanges on the skin. Finally, we compute the stiffener criteria based on the loads in the stiffeners before any failure occurs. In reality, stiffened panels are typically designed to fail progressively, where the skin is allowed to buckle at a subcritical load and the stiffeners are then sized to carry the additional load up to the critical load (Kassapoglou 2013; Iorga et al. 2018; Dähne et al. 2024).

## Aerostructural coupling and derivative computation

To enable coupled analysis and optimization using the capabilities described above, we use the MPhys^12^ (Yildirim et al. 2025) library, which is developed with OpenMDAO^13^ (Gray et al. 2019). OpenMDAO is an open-source Python tool that simplifies the process of building and running complex multidisciplinary computational models. The tool allows users to define coupled models as a series of “components" and the connections between their inputs and outputs. OpenMDAO then automatically coordinates the execution of these components to both solve coupled analyses and, crucially for optimization, compute coupled derivatives, regardless of the number and arrangement of components in the model (Hwang and Martins 2018). MPhys is a library that standardizes how high-fidelity CFD, FE, and other physics codes interface with OpenMDAO, along with a series of “scenario” helper classes that automate the creation of specific standard coupled models. MPhys allows users to quickly build and run complex multidisciplinary models and change the tool used for a given discipline with minimal changes to the setup of their models. TACS, along with the computational tools described below, have existing MPhys interfaces that we use in this work.

### Aerodynamic analysis

For aerodynamic analysis, we use ADflow,^14^ a finite-volume CFD solver for structured multiblock and overset meshes (Mader et al. 2020). ADflow solves the compressible Euler, laminar Navier–Stokes, and RANS equations with a second-order accurate spatial discretization. In this work, we solve the RANS equations with the QCR2000 variant of the Spalart–Allmaras turbulence model. ADflow’s solver employs a variety of numerical methods to converge to a steady-state solution robustly and efficiently, including multigrid, approximate Newton–Krylov, and Newton–Krylov algorithms (Yildirim et al. 2019). The combination of these iterative methods makes ADflow fast and robust. ADflow also solves the discrete adjoint equations, enabling efficient computation of derivatives independent of the number of design variables. The discrete adjoint solution in ADflow relies on the ADjoint approach, which uses algorithmic differentiation (AD) to compute partial derivatives and a Krylov method to solve the adjoint linear system (Kenway et al. 2019).

### Geometrically nonlinear load and displacement transfer

Structural models of wings often use beam elements or represent only the primary wingbox, excluding leading and trailing edge structures as in this work. In these cases, the aerodynamic and structural meshes are non-coincident. This mismatch complicates load and displacement transfer because the displacement of non-coincident parts of the aerodyanmic surface must be extrapolated (rather than merely interpolated) from the structure, and aerodynamic forces on these surfaces induce moments on the structure.

Accurate aerostructural coupling under large deformations requires two additional properties in the load and displacement transfer scheme beyond the standard requirements of consistency and conservation:

1. Displacement extrapolation to offset aerodynamic nodes must be done using a geometrically nonlinear rotation treatment.
2. The moments induced on the structure by the aerodynamic forces must be computed based on the deformed, rather than undeformed configuration.

**Fig. 7:**
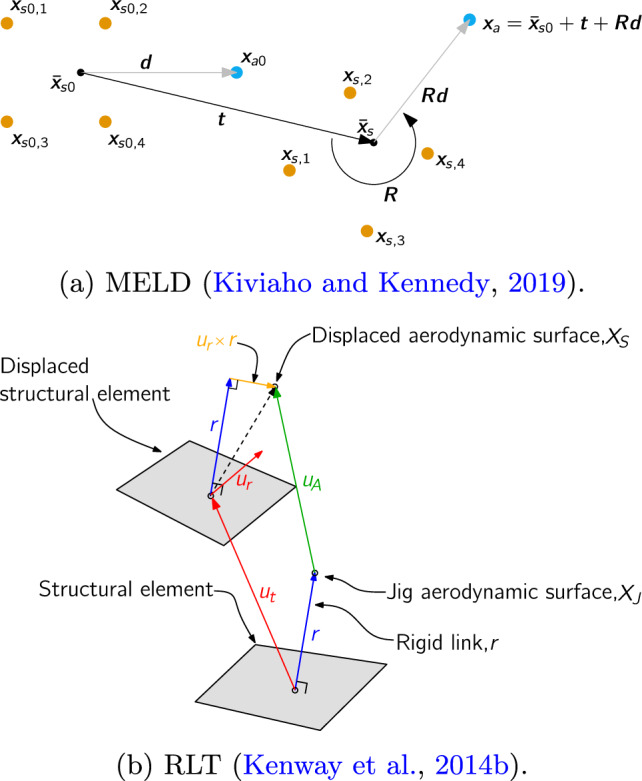
MELD uses a geometrically exact rotation term ($\boldsymbol{Rd}$) when transferring displacements, while RLT uses a linearized approximation ($\boldsymbol{u}_r \times \boldsymbol{r}$)

Most load and displacement transfer schemes, including the rigid link transfer (RLT) scheme used in our previous work and other nonlinear beam studies (Conlan-Smith and Andreasen 2022), use linear transfer operators and satisfy neither requirement. These schemes use linear rotation approximations (e.g., the $u_r \times r$ term in Fig. 7b) and compute loads based on undeformed mesh configurations. This produces nonphysical deformed aerodynamic surface geometry under large twisting rotations and inaccurate structural moments from forces acting on the aerodynamic mesh.

In this work, we use MELD (matching-based extrapolation of loads and displacements), which is a part of the FUNtoFEM framework^15^ (Jacobson et al. 2018). Like RLT, MELD computes aerodynamic node displacements using a fictitious rigid link between meshes. In MELD, this link connects each aerodynamic node to the weighted centroid of the *N* nearest structural nodes, with weights inversely proportional to distance. The centroid displacement is the weighted average of structural node displacements and is transferred directly to the aerodynamic node. Then, crucially, the displacement of the aerodynamic node due to the rotation of the structural mesh is computed by finding the rotation matrix that best recovers the observed structural displacements (Kiviaho and Kennedy 2019). Large rigid rotations of the structure are therefore transferred exactly to the aerodynamic mesh.

To make MELD a conservative scheme, the load transfer operator is derived from the virtual work principle:

21 $$\begin{aligned} \frac{\partial }{\partial \boldsymbol{u}_s}\left( \boldsymbol{u}_s^\text {T}\boldsymbol{f}_s\right)&= \frac{\partial }{\partial \boldsymbol{u}_s}\left( \boldsymbol{u}_a^\text {T}\boldsymbol{f}_a\right) \end{aligned}$$

22 $$\begin{aligned} \frac{\partial }{\partial \boldsymbol{u}_s}\left( \boldsymbol{u}_s^\text {T}T_f\left( \boldsymbol{f}_a\right) \right)&= \frac{\partial }{\partial \boldsymbol{u}_s}\left( T_u\left( \boldsymbol{u}_s\right) ^\text {T}\boldsymbol{f}_a\right) \end{aligned}$$

23 $$\begin{aligned} \boldsymbol{f}_s = T_f\left( \boldsymbol{f}_a\right)&= \frac{\partial T_u\left( \boldsymbol{u}_s\right) }{\partial \boldsymbol{u}_s}^\text {T}\boldsymbol{f}_a, \end{aligned}$$

where $\boldsymbol{u}_s$ and $\boldsymbol{u}_a$ are the structural and aerodynamic displacements, respectively, $\boldsymbol{f}_s$ and $\boldsymbol{f}_a$ are the structural and aerodynamic forces, respectively, $T_f$ is the load transfer operator, and $T_u$ is the displacement transfer operator. Because the displacement transfer operation is nonlinear, the load transfer operator is also nonlinear and correctly accounts for the deformed configurations of both meshes. Because MELD uses only nodal displacements from the structure, the moments resulting from non-coincident meshes are resolved as nodal forces rather than point moments (as in RLT). This approach is more physically realistic. The computational cost of the MELD scheme is higher than of RLT because each aerodynamic node is connected to a larger number of structural nodes, and the operations involved in the load and displacements transfer are more complex. However, the cost of both schemes is negligible compared to the cost of the aerodynamic and structural solutions involved in the coupled analysis process.

### Geometry parameterization

To parameterize the geometry of the aerodynamic and structural models during optimizations, we use the free-form deformation (FFD) implementation in pyGeo^16^ (Hajdik et al. 2023). This approach embeds the mesh nodes of a model in a volume defined by a set of control points. Deformations at the control points are then smoothly mapped to the embedded points using spline-based interpolation. In this way, point sets from multiple models are deformed consistently, which is critical in the case of aerostructural optimization where the aerodynamic and structural geometries should remain coincident as they change. The derivatives of the embedded points’ coordinates with respect to user-defined design variables that dictate the control point deformations can be computed quickly and accurately because the mapping is analytic (Kenway et al. 2010).

### Coupled solvers

MPhys allows coupled models to be solved using any of the linear and nonlinear solvers available in OpenMDAO. For primal aerostructural analyses, we use a nonlinear block Gauss–Seidel (NLBGS) solver, which is a partitioned approach commonly used for solving coupled aerostructural problems (Kenway et al. 2014b; Gray and Martins 2021; Martins and Ning 2022, Sec. 13.2.5). In each cycle of the nonlinear solution, the structural model is converged tightly, while the aerodynamic model is only converged loosely. The solver is stabilized using Aitken acceleration (Irons and Tuck 1969). For the coupled adjoint solution, we use the FGMRES iterative solver (Saad 1993) to converge the monolithic coupled linear system. We precondition each FGMRES iteration with one round of linear block Gauss–Seidel, which involves solving the single discipline adjoints in TACS and ADflow approximately.

Switching from a linear to a nonlinear structural model introduces an additional challenge in the coupled analysis: the structural solver may fail to converge for a given aerodynamic load.^17^ We have found these solution failures to be rare in our optimizations, likely because the conservative nature of our buckling failure criteria and our constraint that the wing have at least a safety factor of 1.5 against buckling failure dissuade the optimizer from evaluating designs in which the shell FE model itself would buckle and fail to converge. Nevertheless, handling these solver failures robustly is critical in any CFD-based optimization, and we extend the same approach to the structural solver. In cases where the aerodynamic or structural solver reaches its maximum number of iterations without reaching the desired convergence tolerance, we simply continue the NLBGS iterations. The failing disciplinary solver, and thus the coupled solver, often eventually converges as the changes in the coupling variables (aero forces and structural displacements) between NLBGS iterations reduce. In cases where the NLBGS solver reaches its iteration limit without converging, or a fatal failure occurs in the solver (e.g., a NaN value in the solution or negative volumes in the CFD mesh), we return a flag to the optimizer indicating that the objective and constraints are undefined at the current design point. The optimizer then backtracks until it finds a design point where the coupled solver can converge and continues from there. Provided these solution failures do not occur at the eventual optimum, they do not adversely affect the convergence of the optimization.

### Optimizer

To perform our optimizations, we use the sparse nonlinear optimizer, SNOPT (Gill et al. 2005), a high-performance sequential quadratic programming (SQP) algorithm that has proven to excel at the large-scale, sparse, constrained nonlinear optimization problems typical of high-fidelity MDO (Lyu et al. 2014). OpenMDAO interfaces with SNOPT through pyOptSparse^18^ (Wu et al. 2020), an object-oriented framework that provides a general interface to many optimizers and simplifies the task of defining large sparse Jacobians that are crucial for the performance of large-scale optimizers like SNOPT.

## Aerostructural optimization problem

We demonstrate our framework’s capabilities using the simple transonic wing (STW) benchmark model.^19^ The model geometry, mission profile, and flight conditions are based on the Boeing 717, representing a single-aisle commercial jet aircraft. While geometrically nonlinear aeroelastic phenomena are typically associated with experimental aircraft such as NASA’s Helios prototype, modern commercial aircraft like the Boeing 787 already exhibit deflections beyond 20% semispan (Dodt 2011). This, combined with the necessity of RANS CFD to accurately capture transonic flow over their wings, makes commercial jet aircraft an appropriate test case for our framework.

### Wing geometry

**Fig. 8:**
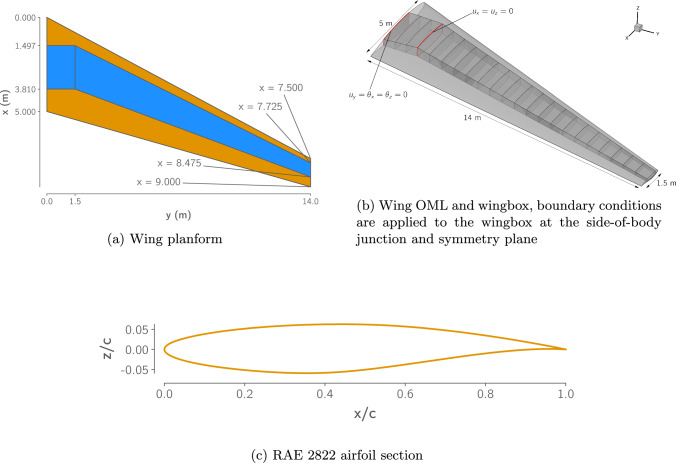
Simple transonic wing OML and wingbox geometries

The STW geometry is shown in Fig. 8. It has a simple trapezoidal planform, with a constant, untwisted RAE 2822 cross-section. The wing contains a conformal wingbox with upper and lower skins, leading and trailing edge spars, and 23 ribs. The wingbox features a typical side of body (SOB) break at a semispan of 1.5 m, inboard of which the wingbox is unswept, resembling a center-box. Outboard of the SOB, the wingbox extends from 15 to 65% of the chord. The wingbox is subject to symmetry conditions at the centerline and is fixed in the chordwise and vertical directions at the SOB as shown in Fig. 8b.

These boundary conditions capture the primary load transfer mechanisms between the wing and the fuselage. Vertical and chordwise shear forces are transferred into the fuselage frame at the SOB, while the axial forces caused by wing bending are transferred into the center wingbox. Applying these boundary conditions rigidly at each section leads to inaccurate reaction forces and thus inaccurate stresses near the boundaries. The exact distribution of the reaction forces at these boundaries cannot be accurately captured without explicitly modeling the fuselage structure, or at least approximating its stiffness. However, this boundary condition simplification is consistent with the level of detail with which we model and optimize the wingbox.

### Aerodynamic model

**Fig. 9:**
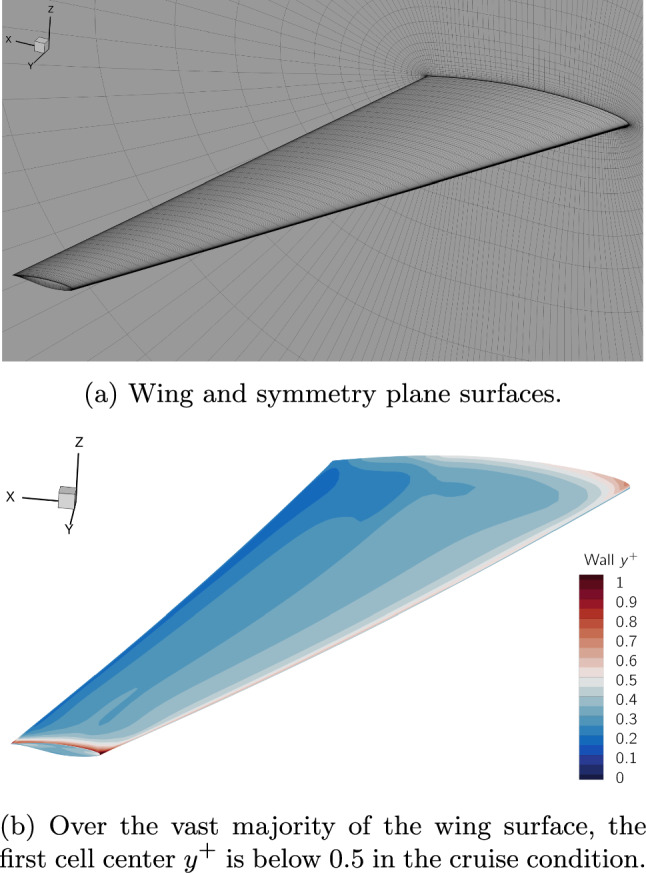
The $9.7 \times 10^5$ cell multiblock CFD mesh used in this work

We use the L2 multiblock structured CFD mesh provided in the benchmark repository and shown in Fig. 9. The mesh contains 970,000 cells and has a $y^+$ below 0.5 over the vast majority of the wing surface in the cruise condition.

### Structural model

**Fig. 10:**
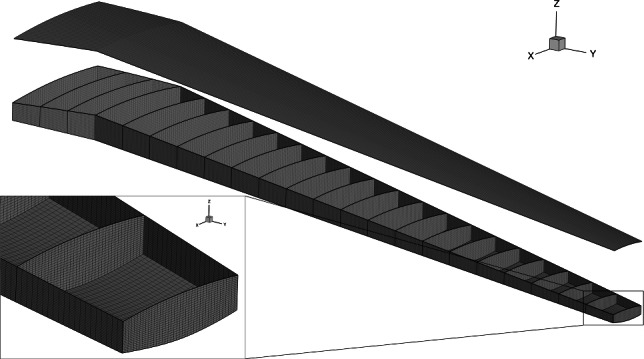
The wingbox mesh contains 71,200 quadrilateral elements and 419,778 DOFs

The wingbox is composed of stiffened composite panels, modeled using the stiffened shell constitutive model described in Sect. 2.3. The stiffeners are assumed to have a T-shaped cross-section. The unidirectional composite ply properties used throughout the wingbox are shown in Table 2, taken from Brooks et al. (2020). Both the shell and stiffeners in every wingbox panel are assumed to consist of $[0,\,-45,\,+45,\,90]^{\circ }$ plies. Different smeared laminates are used for different components in the wingbox based on values used by Dillinger (2014). In the upper and lower skin shells and in all stiffeners, we assume a ${0}^{\circ }$ biased layup with ply fractions of [44.41%, 22.2%, 22.2%, 11.19%], while in the spar and rib shells, we use a more isotropic [10%, 35%, 35%, 20%] laminate. In the skins, the stiffeners and ${0}^{\circ }$ plies are aligned with the trailing edge spar; in the spars and ribs, they are vertically oriented (Fig. 10).

**Table 2:** Composite ply properties

Property	Description	Value
$E_{11}$	Longitudinal modulus	117.7 GPa
$E_{22}$	Transverse modulus	9.7 GPa
$G_{12}$	In-plane shear modulus	4.8 GPa
$G_{13}$	Transverse shear modulus	4.8 GPa
$G_{23}$	Transverse shear modulus	4.8 GPa
$T_1$	Longitudinal tensile strength	1648 MPa
$C_1$	Longitudinal compressive strength	1034 MPa
$T_2$	Transverse tensile strength	64 MPa
$C_2$	Transverse compressive strength	228 MPa
$S_{12}$	Shear strength	71 MPa
$\nu _{12}$	Major Poisson’s ratio	0.35
$\rho$	Density	1550 kg $\textrm{m}^{-3}$

### Optimization problem

We solve the third benchmark optimization problem posed for the STW. The full problem is summarized in Tables 3, 4 and 5.

**Table 3:** Flight conditions

Flight point	Altitude	Mach number	Load factor	Aircraft mass
Cruise	10400 m	0.770	1	$\sqrt{M_\text {cruise, start}\times LGM}$
Pull-up maneuver	0 m	0.458	2.5	*LGM*
Push-down maneuver	0 m	0.458	$-1$	*LGM*

**Table 4:** Aircraft and mission specifications, based on the Boeing 717 high gross-weight variant

	Quantity	Description	Value
*Baseline wing geometry*			
	*b*	Semispan	14 m
	$C_\text {root}$	Root chord	5 m
	$C_\text {tip}$	Tip chord	1.5 m
	*S*/2	Planform area (single wing)	45.5 m^2^
	$\text {MAC}$	Mean aerodynamic chord	3.56 m
*Masses*			
	$M_\text {payload}$	Payload mass	$14.5\,\times \,10^{3}$ kg
	$M_\text {frame}$	Operating empty mass (excluding wing)	$25\,\times \,10^{3}$ kg^a^
	$M_\text {fuel, res}$	Reserve fuel mass	$2\,\times \,10^{3}$ kg
*Fuelburn calculation parameters*			
	*R*	Nominal range	3815 km
	$R_\text {climb}$	Climb segment range	290 km
	$V_\text {climb}$	Average climb speed	350mph
	$C_{D,\text {frame}}$	Airframe drag coefficient (fuselage + tail + nacelle)	0.01508^b^
	$k_\text {tank}$	Assumed fraction of wingbox that can store fuel	0.85
	$V_\text {aux}$	Auxilliary fuel tank volume	2.763 m^3^
	$\text {TSFC}$	Thrust specific fuel consumption	$18\, \times \, 10^{-6}$ kg $\textrm{N}^{-1}$ s^c^
	$\rho _\text {fuel}$	Fuel density	804 kg $\textrm{m}^{-3}$

^a^ Approximated based on true empty weight of $31.1\,\times \,10^{3}$ kg

^b^ Computed using a conceptual design drag build-up

^c^ Publicly available value for the Rolls-Royce BR715 engine (Roux 2007)

**Table 5:** Full optimization problem specification

	Function/Variable	Description	Quantity
*Minimize*	FB	Fuel burn	1
*By varying*	$t_{\text {sh}}$	Shell thicknesses	111
	$t_{\text {st}}$	Stiffener thicknesses	111
	$h_{\text {st}}$	Stiffener heights	111
	$\tilde{L}$	Panel lengths	111
	$\alpha _\text {2.5g}$	Pull-up maneuver angle of attack	1
	$\alpha _\text {-1g}$	Push-down maneuver angle of attack	1
	$\alpha _\text {cruise}$	Cruise angle of attack	1
	$\theta$	Section twists	5
	$\Delta z_\text {local}$	Section shape perturbations	91
	$x_\text {chord}$	Chord scaling	2
	$\Delta y_\text {tip}$	Span change	1
	$\Delta x_\text {tip}$	Tip shear	1
		**Total**	**547**
*Such that*	$1.5 SR_\text {2.5g} \le 1$	Pull-up maneuver strength ratio	4
	$1.5 SR_\text {-1g} \le 1$	Push-down maneuver strength ratio	1
	$\left| t_{\text {sh},i} - t_{\text {sh},j}\right| \le$ 2.5 mm	Skin/spar shell thickness adjacency	84
	$\left| t_{\text {st},i} - t_{\text {st},j}\right| \le$ 2.5 mm	Skin/spar stiffener thickness adjacency	84
	$\left| h_{\text {st},i} - h_{\text {st},j}\right| \le$ 10 mm	Skin/spar stiffener height adjacency	84
	$t_{\text {st},i} \le 15 t_{\text {sh},i}$	Maximum stiffener thickness	111
	$h_{\text {st},i} \le 30 t_{\text {st},i}$	Maximum stiffener aspect-ratio	111
	$h_{\text {st},i} \ge 5 t_{\text {st},i}$	Minimum stiffener aspect-ratio	111
	$w_{\text {st},i} \le p_{\text {st},i}$	Minimum stiffener spacing	111
	$\tilde{L}_{i} = L_{i}$	Panel length consistency	111
	$L_\text {2.5g} = 2.5 LGM g$	Pull-up maneuver lift level	1
	$L_\text {-1g} = -LGM g$	Push-down maneuver lift level	1
	$L_\text {cruise} = M_\text {mid-cruise} g$	Cruise maneuver lift level	1
	$t_\text {spar} \ge 0.75 t_{\text {spar},0}$	Minimum spar height	40
	$t \ge 0.5 t_{0}$	Minimum TE thickness	400
	$R_\text {LE} \ge 0.9 R_{\text {LE},0}$	Minimum LE radius	20
	$M_\text {fuel}/\rho _\text {fuel} \le V_\text {aux} + 2k_\text {tank} V_\text {wingbox}$	Fuel volume	1
	$TOGM / S \le$ 600 kgm$^{-1}$^2^	Maximum wing loading	1
		**Total**	**1277**

#### Objective

The optimization problem minimizes the aircraft’s fuel burn over a given mission, which is based on the wing’s lift-to-drag ratio in the cruise condition detailed in Table 3. We compute the cruise lift and drag using a coupled aerostructural analysis in MPhys and an assumed drag coefficient for the components of the aircraft other than the wing.

#### Design variables

**Fig. 11:**
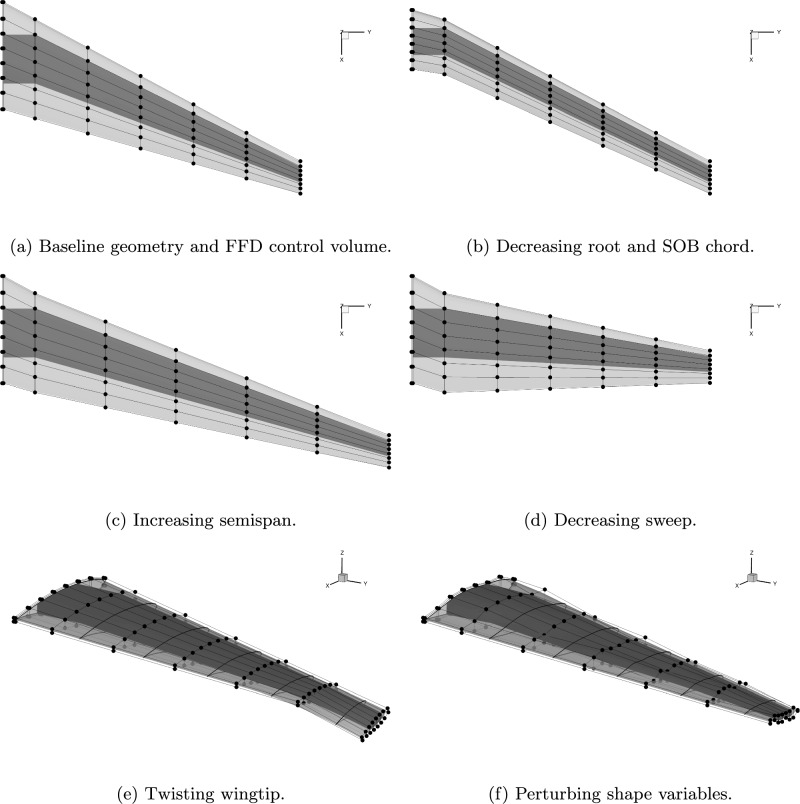
Geometric design variables

We use 544 structural sizing and geometric design variables to allow the optimizer to trade-off the wing’s aerodynamic and structural performance. Figure 11 shows the OML and wingbox inside the FFD control volume that parametrizes the geometry. This control volume has seven spanwise stations, one at the symmetry plane, one at the SOB junction, and five more evenly spaced along the remaining span. Each of these sections has 8 control points in the chordwise direction on both the upper and lower surfaces. We control the twist, shape, chord, span, and sweep of wing using the FFD volume as follows:

*Twist*: We hold the wing root twist angle fixed as the wing’s angle of attack is a design variable. We also fix the twist at the SOB to avoid an unrealistic center wingbox geometry. The twist of each of the remaining five FFD stations about their leading edge is a separate design variable.

*Shape*: We parameterize section shapes through the vertical motion of the FFD control points. The upper and lower control points at the trailing edge of each section are fixed to avoid pinching the blunt trailing edge. At the leading edge, the upper and lower control points are forced to move together in opposite directions so that the shape variables cannot modify the section twist angle.

*Chord*: The chord distribution is parameterized by two design variables, the first variable scales the chord at the wing root and SOB, the second scales the chord at the wing tip. The chord length is then linearly interpolated between the SOB and tip. Scaling the chord at the root and SOB by the same factor is necessary to avoid tapering the center wingbox. We also shift the root station in the chordwise direction to keep the center wingbox unswept.

*Span and Sweep*: The span and sweep are each controlled by single design variables that specify the wingtip’s spanwise and chordwise displacement respectively. These displacements are linearly interpolated from the tip to the SOB, which is fixed.

To parameterize the structure, we treat each rib and each skin and spar segment between a pair of ribs as a separate panel. The optimizer can vary the shell thickness, $t_\text {sh}$, stiffener height, $h_\text {st}$, and stiffener thickness, $t_\text {st}$ (see Fig. 6). The stiffener flange fraction, $k_f$, is set at 1 for all panels and the stiffener pitch, $P_\text {st}$ is fixed at 150 mm. An additional panel length variable, $\tilde{L}$ does not affect the stiffness or geometry of each panel but must be passed to each element to compute the buckling failure criteria described in Sect. 2.4. We add a set of constraints to ensure that the panel length design variables are consistent with the true length of each panel.

While our constitutive model allows the ply fractions in the skin and stiffener laminates to be used as design variables, we do not use them in this work for several reasons. First, our analysis focuses on the differences between the optimum designs rather than the differences between the baseline and optimized designs. Investigating such differences between optima requires the optimizations to be converged more tightly than is necessary to produce useful results. Without sufficient convergence, differences between optima could be due to the differences in the models being studied, or simply because the two optimizations were stopped prematurely while taking different paths toward the same optimum. Introducing additional ply fraction design variables would make it harder to achieve this level of convergence. Second, introducing ply fraction variables would require numerous complex constraints to ensure that the optimized laminates are manufacturable from a set of continuous plies (Kennedy and Martins 2013). Including such manufacturability constraints in gradient-based optimizations where the discrete stacking sequence is not defined is a challenging problem and remains an area of active research (Corrado et al. 2022).

Finally, the optimizer can also control the angle of attack for each flight condition to meet the lift constraints described in Sect. 4.4.3.

#### Constraints

The primary structural constraints enforce that the wingbox has a safety factor of 1.5 for both material and buckling failure in both maneuver flight conditions. We evaluate all the failure criteria described in Sect. 2.4 at the integration points in each element in the structural model. We then use multiple levels of KS aggregation (Kreisselmeier and Steinhauser 1979) to reduce the number of failure constraints in the optimization and, thus, the number of expensive coupled adjoint solutions that need to be computed. First, the strength ratios for each failure mode are aggregated into a single value at each integration point. Then, the integration point values are aggregated into a single value for each element. Finally, the element values are aggregated across the wingbox. In this work, we split this final aggregation to produce four strength ratio values, one each for the upper skin, lower skin, spars, and ribs. In the 2.5g maneuver, we constrain all aggregated strength ratios. In the −1 g maneuver, we assume that only the lower skin is failure critical (with respect to buckling) and thus constrain only the lower skin strength ratio to reduce the number of coupled adjoint solutions required in each iteration of the optimization.

We enforce structural adjacency constraints to avoid abrupt changes in panel sizing. The change in shell and stiffener thicknesses between adjacent skin and spar panels is limited to 2.5 mm and the change in stiffener height to 10 mm.^20^ We also enforce the following structural sizing rules suggested by Kassapoglou (2013) on all panels:

- The skin and stiffener thicknesses must be at least 0.6 mm.
- The stiffener heights must be at least 18 mm.
- The stiffener flange widths must be at least 25.4 mm.
- The aspect ratio of the stiffener web ($h_\text {stiff}/t_\text {stiff}$) must be between 5 and 30.
- The thickness of the stiffener flanges on a panel must be no more than 15 times the skin thickness.
- The stiffener flange width must be less than the stiffener pitch to avoid overlapping flanges.

Although numerous, these constraints are linear and handled efficiently by SNOPT, not requiring repeated gradient computations.

We constrain the lift produced by the wing at each flight point to equal the aircraft’s weight multiplied by the relevant load factor. We model the maneuver condition at the landing gross mass because our structural model does not include the fuel’s inertial relief. The aircraft mass for the cruise condition is assumed to be the mid-cruise mass, which we compute using a geometric average of the mass at the start and end of the cruise segment to account for the non-uniform rate of fuel burn over the segment.

**Fig. 12:**
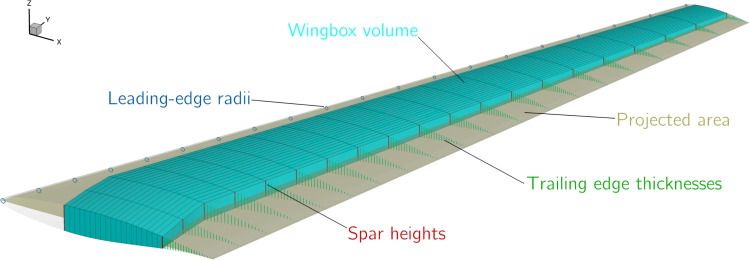
Geometric constraints ensure that the optimization produces a realistic geometry

Since we only consider three flight points, additional constraints are required to ensure the optimizer produces a realistic wing geometry, these are shown in Fig. 12. To maintain reasonable low-speed performance, we enforce that the leading edge radius of the wing remains at least 90% of the baseline value at 20 points along the span, we also compute the projected planform area of the wing and use it to enforce a maximum allowable wing loading ($\text {TOGM}/2S$) of 600kg $m^{-2}$. We compute the volume of the wingbox and enforce that it is large enough to store the fuel required for the mission, assuming that fuel tanks make up 85% of the wingbox. The heights of the front and rear spars at 20 points along the span are constrained to be at least 75% its original value to maintain the space required to mount components such as control surface actuators (Liem et al. 2015). Finally, the region between the rear spar and the trailing edge is constrained to maintain at least 50% of its original thickness to prevent the optimizer from producing an unrealistically thin trailing edge, which is a common issue in aerodynamic shape optimization.

## Results

In this section, we compare solutions to the above optimization problem computed using both linear and geometrically nonlinear structural formulations. We vary the load and displacement transfer scheme to match the structural formulation, using the linearized form of MELD for the linear structural model and the exact form for the geometrically nonlinear model^21^.

Since we are primarily concerned with the impact of geometric nonlinearity on the optimization results, we focus our analysis on the differences between the two optimized designs rather than on the differences between the baseline and optimized designs. We include a more detailed analysis of the linear optimization results in previous work (Gray and Martins 2025).

Beyond comparing the performance of the two designs using their respective structural formulations, we examine the performance degradation of the wing designed with the linear structural model when operating in the real, geometrically nonlinear, world. Optimizing using the geometrically nonlinear structural formulation, while more expensive, promises to yield designs that perform as expected when built. To investigate this, we analyze the linearly optimized wing using the nonlinear structural formulation. This analysis includes re-trimming the wing to the correct load factor at each flight point and accounting for fuel mass changes due to drag variations. These results are labeled “Linear Re-analyzed” in the tables and figures below.

We do not consider results from this single optimization problem sufficient to draw general conclusions about the impact of geometric nonlinearity on HARW design. A more rigorous study of the impact of geometric nonlinearity on optimal HARW design needs further study.

### Derivative verification

Since accurate derivatives are crucial for solving such large-scale aerostructural optimization problems, we first verify the accuracy of the coupled adjoint derivatives computed by MPhys. To do this, we compute the derivatives of an aerodynamic ($C_L$) and structural (lower skin strength ratio) function with respect to a geometric (wing sweep) and an aerodynamic (angle of attack) variable, and a random perturbation of the structural sizing variables. Table 6 compares these derivatives to values computed using (central) finite differences. All values are computed on the two optimized designs presented later in this section in the 2.5g maneuver condition. The largest error between the coupled adjoint and finite difference derivatives is 0.03% while the lowest is 0.00005%, these are similar orders of magnitude to those reported by Kenway et al. (2014b) when comparing against more accurate complex-step approximations. Both the linear and nonlinear formulations achieve similar accuracy.

**Table 6:** The coupled adjoint derivatives for both the linear and nonlinear formulations are accurate

		${\frac{d C_L}{d x_\text {sweep}}}$	${\frac{d C_L}{d x_\text {struct}}}$	${\frac{d C_L}{d \alpha }}$	${\frac{d SR}{d x_\text {sweep}}}$	${\frac{d SR}{d x_\text {struct}}}$	${\frac{d SR}{d x_\alpha }}$
Linear	Coupled adjoint	$\boldsymbol{8.246}643\times 10^{-2}$	$\boldsymbol{-7.03142}1\times 10^{-2}$	$\boldsymbol{2.56}4581\times 10^{-2}$	$-\boldsymbol{4.89}5928\times 10^{-2}$	$\boldsymbol{1.008}706\times 10^{-1}$	$\boldsymbol{1.30527}1\times 10^{3}$
	Finite difference	$\boldsymbol{8.246}056\times 10^{-2}$	$\boldsymbol{-7.03141}8\times 10^{-2}$	$\boldsymbol{2.56}4462\times 10^{-2}$	$-\boldsymbol{4.89}7246\times 10^{-2}$	$\boldsymbol{1.008}962\times 10^{-1}$	$\boldsymbol{1.30527}2\times 10^{3}$
	Error (%)	$7.1\times 10^{-3}$	$5.4\times 10^{-5}$	$4.6\times 10^{-3}$	$-2.7\times 10^{-2}$	$-2.5\times 10^{-2}$	$-8.2\times 10^{-5}$
Nonlinear	Coupled adjoint	$\boldsymbol{5.827}184\times 10^{-2}$	$\boldsymbol{-6.3478}27\times 10^{-2}$	$\boldsymbol{1.9590}76\times 10^{-2}$	$\boldsymbol{1.3890}06\times 10^{-1}$	$\boldsymbol{-2.225}494\times 10^{-2}$	$\boldsymbol{-1.47277}6\times 10^{3}$
	Finite difference	$\boldsymbol{5.827}351\times 10^{-2}$	$\boldsymbol{-6.3478}91\times 10^{-2}$	$\boldsymbol{1.9590}08\times 10^{-2}$	$\boldsymbol{1.3889}99\times 10^{-1}$	$\boldsymbol{-2.225}207\times 10^{-2}$	$\boldsymbol{-1.47277}8\times 10^{3}$
	Error (%)	$-2.9\times 10^{-3}$	$-1.0\times 10^{-3}$	$3.5\times 10^{-3}$	$4.9\times 10^{-4}$	$1.3\times 10^{-2}$	$-1.5\times 10^{-4}$

Bold digits indicate the significant digits to which the coupled adjoint and finite difference values match

### Optimization results

**Fig. 13:**
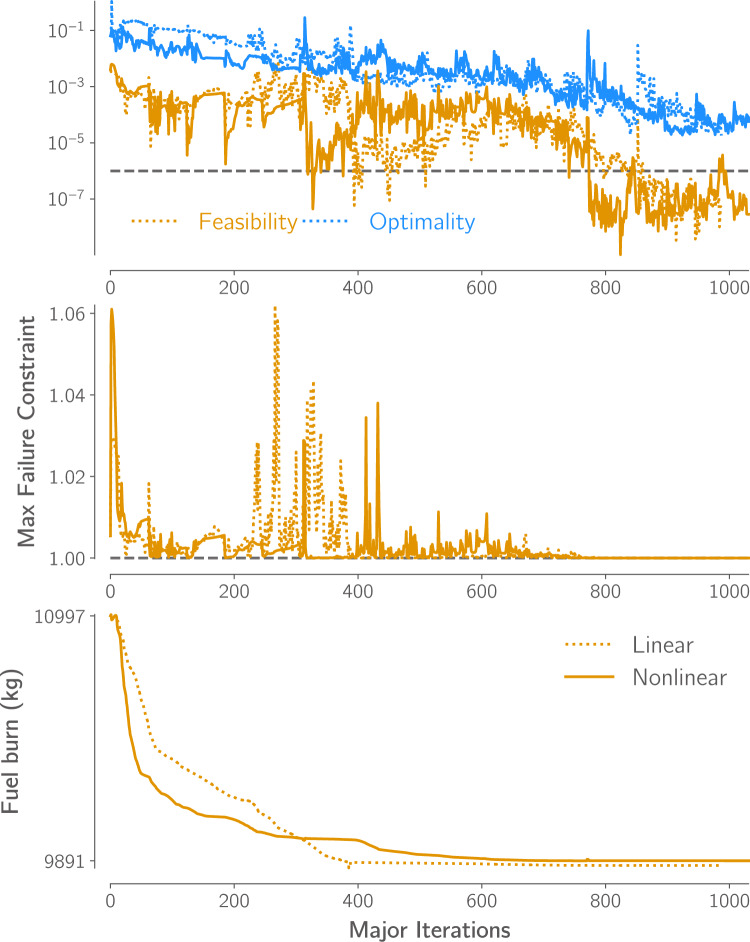
Both optimizations produce feasible designs and reduce first-order optimality by over three orders of magnitude

Figure 13 shows the convergence history of the two optimizations. Both optimizations ran on 320 Intel Xeon Gold 6248 or 6148 cores. The optimization using the linear structural formulation took 110.6 h, while the optimization using the geometrically nonlinear structural formulation took 189.6 h.^22^ In this time, SNOPT completed approximately 1000 major iterations,^23^ achieved the desired feasibility^24^ tolerance of $10^{-6}$, and reduced the optimality measure^25^ by over three orders of magnitude in both cases. Tightly converging optimality in such large-scale aerostructural optimization problems is challenging due to the limited accuracy of the coupled aerostructural derivatives caused by the structural model’s poor conditioning (Kenway et al. 2014b).

**Fig. 14:**
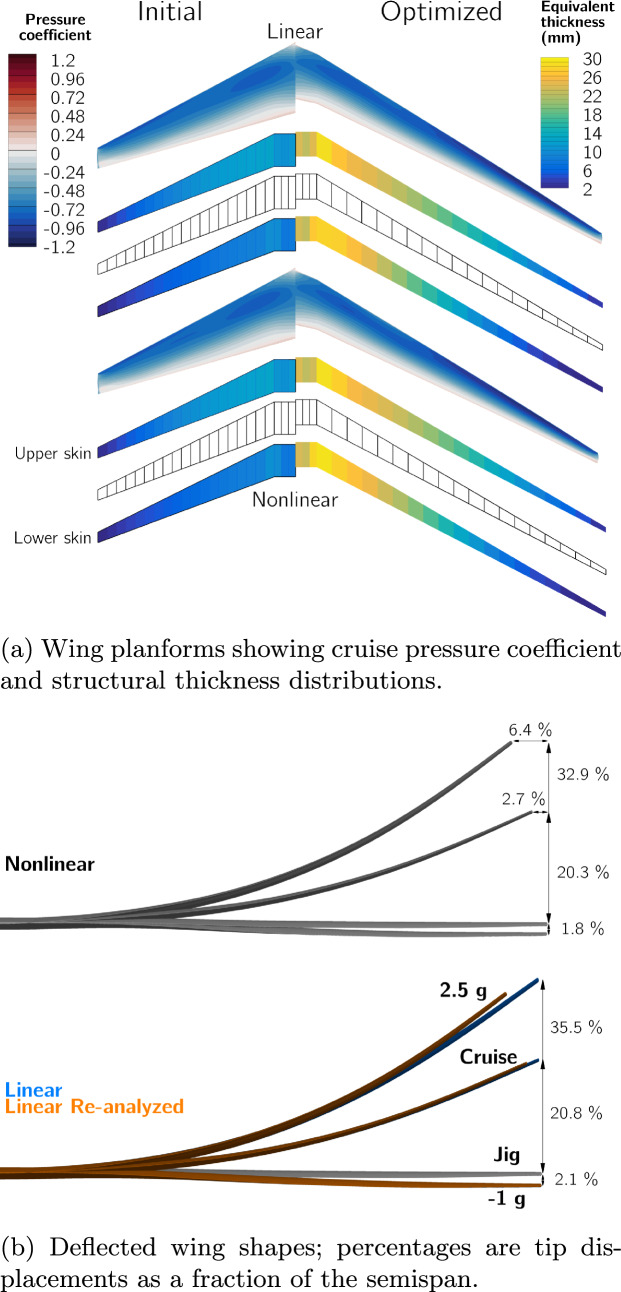
Comparison of the wings optimized with linear and nonlinear formulations

Figure 14a shows the two optimized designs,^26^ and Table 7 lists key quantities of interest for both, and for the re-analyzed linear design. The planforms of the two optimized designs are very similar, the optimizer increases the wingspan and reduces the root and tip chord lengths, more than doubling the aspect ratio from 8.6 to over 19. The nonlinear design has a 1% higher span and 1.6% higher aspect ratio. This leads to a 0.2% increase in *L*/*D* but also a 0.4% increase in empty weight and 0.2% increase in fuel burn.

Both designs have tip displacements of over 20% of their semispan in cruise and over 30% in the 2.5g maneuver. When analyzed with the linear formulation, the wing’s projected span remains constant, meaning that the bending deflection artificially increases its total length and area, leading to larger tip displacements. However, when the linear design is re-analyzed with the nonlinear formulation, the tip displacements are reduced, matching those of the nonlinear design, and the projected span is reduced by 2.6% in cruise. Despite the loss in span, the decrease in overall aircraft *L*/*D* and in crease in fuel burn is only 0.15%. This is likely because a reduction in skin-friction due to the wing’s smaller wetted area offsets the increase in induced drag caused by the span reduction.

**Fig. 15:**
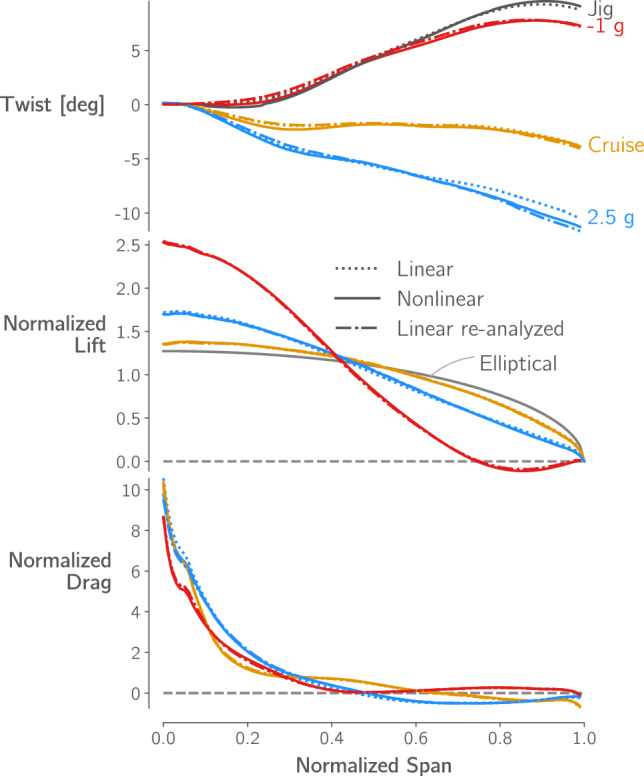
Twist and lift distributions of the two optimized designs and the linear optimized design re-analyzed with nonlinear analysis

Figure 15 shows the twist, lift, and drag distributions of the two designs and the re-analyzed linear design. The largest difference in jig twist distributions of the two optimized designs occurs between the SOB and 50% span, where the nonlinear design increased the washout by ${0.5}^{\circ }$. The cruise and $-1g$ in-flight twist distributions differ by similarly small amounts, but the 2.5g condition shows more significant differences. When the linear design is re-analyzed with the nonlinear formulation, it achieves less washout in the inboard section of the wing but more washout toward the tip. Specifically, the tip washout of linear design increases from ${-10.5}^{\circ }$ to ${-11.6}^{\circ }$. This may be due to the nonlinear drag-torsion coupling discussed by Garcia (2005), except that, where Garcia showed a wash-in torsion caused by drag, here we see a washout torsion caused by the thrust produced by the outer half of the wing (shown in the bottom subplot of Fig. 15). Despite these differences, the differences in the lift distributions and the amount of passive load alleviation achieved in each flight by each design condition are small and likely insignificant. This is true both when comparing the two optimized designs and when comparing the linear and nonlinear analyses of the linear design.

**Table 7:** Quantities of interest for the optimized wings

Quantity	Linear	Nonlinear	Linear re-analyzed	Linear re-sized	Units
$M_\text {wingbox}$	1528.50	1580.31	1528.50	1580.82	kg
$M_\text {wing}$	4030.27	4141.44	4030.27	4142.53	kg
LGM	49560.55	49782.87	49560.55	49785.06	kg
TOGM	59430.82	59674.16	59446.37	59682.08	kg
$M_\text {fuel}$	9870.27	9891.29	9885.83	9897.01	kg
$\alpha _\text {cruise}$	3.81	3.91	3.88	3.88	$^{\circ }$
$\alpha _\text {2.5g}$	10.68	11.16	11.30	11.31	$^{\circ }$
$\alpha _\text {-1g}$	$-$7.63	$-$7.44	$-$7.63	$-$7.65	$^{\circ }$
Cruise *L*/*D*	19.35	19.39	19.32	19.39	
Fuel tank usage	100.00	99.75	100.16	100.28	%
Wing semispan	21.81	22.04	21.81	21.81	m
Wing aspect ratio	19.21	19.53	19.21	19.21	
Wing root chord	3.84	3.86	3.84	3.84	m
Wing tip chord	0.78	0.74	0.78	0.78	m
Taper ratio	0.20	0.19	0.20	0.2	
Wing leading edge sweep	31.52	31.12	31.52	31.52	$^{\circ }$
Wing area	49.53	49.73	49.53	49.53	$\textrm{m}^{2}$
Wing loading	600.00	600.00	600.16	602.54	kg $\textrm{m}^{-2}$
2.5 g Lower skin FOS	1.50	1.50	1.12	1.50	
2.5 g Upper skin FOS	1.50	1.50	1.34	1.50	
2.5 g Spars FOS	1.50	1.50	0.62	1.50	
2.5 g Ribs FOS	1.50	1.50	0.85	1.50	
-1 g Lower skin FOS	1.50	1.50	1.51	1.50	

FOS = Factor of safety

**Fig. 16:**
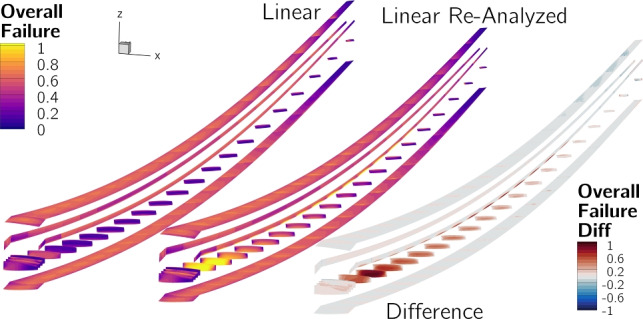
The wing optimized with the linear structural formulation is structurally infeasible when re-analyzed with the nonlinear formulation

So far, we have shown that geometrically nonlinear wing deformations do not significantly change static aerodynamic performance or loads. However, as discussed in our introduction, even under similar loading, geometrically nonlinear effects can significantly alter the distribution of internal forces in a structure. To investigate this effect, Fig. 16 shows the overall strength ratio distribution in the linear wingbox design in the 2.5g maneuver condition. The leftmost wingbox shows results from the linear analysis, the middle wingbox shows results from the nonlinear analysis, and the rightmost wingbox shows the difference between the two. These results show that the wing designed using linear analysis is structurally infeasible.

**Fig. 17:**
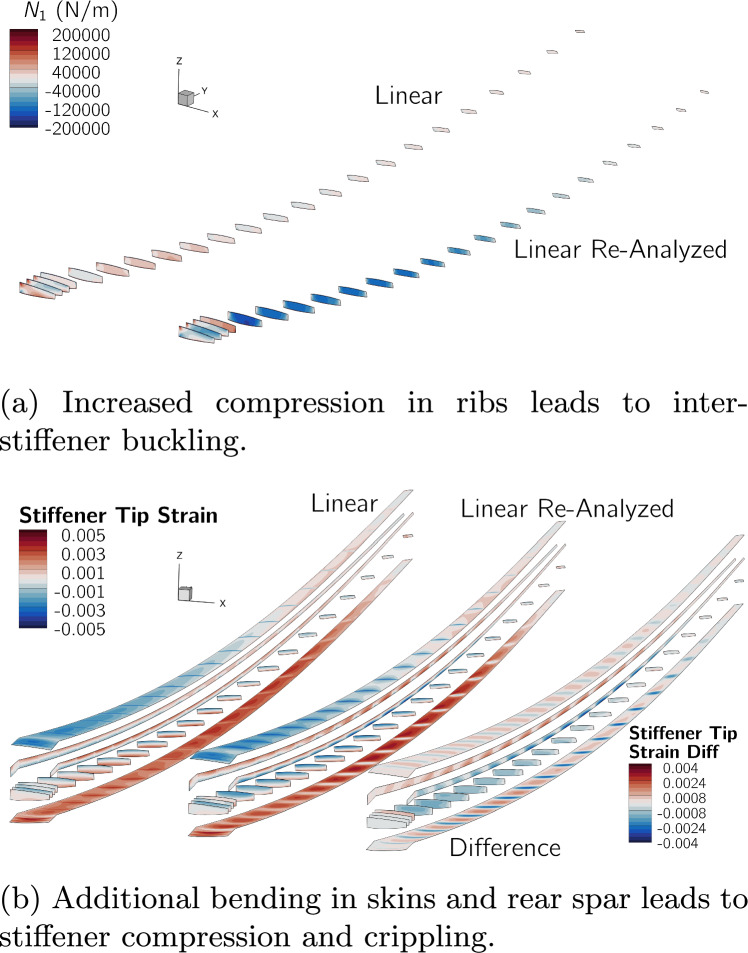
Brazier effects cause buckling in ribs and stiffener crippling in the skins and rear spar

The cause of this infeasibility is the Brazier effect. As previously mentioned, this is the tendency for the cross-section of a beam to flatten under bending. The wingbox ribs resist this flattening and thus experience crushing loads, which are not captured by the linear analysis, as shown in Fig. 17a. In this case, these crushing loads in the ribs near the SOB cause the factor of safety to inter-stiffener buckling to be as low as 0.85.

Between ribs, where the skins are not vertically supported, the wingbox section does flatten. This variation in the amount of cross-section flattening between supported and unsupported sections leads to local bending moments in the wing skins around each rib which, as shown in Fig. 17b, are large enough to cause compressive strains in the lower skin stiffeners, reducing the factor of safety to stiffener crippling to 1.12. Finally, where the section does flatten, significant bending is induced in the front and rear spars. In our model, the stiffeners are assumed to be on the front side of the spars, meaning that the stiffeners on the rear spar are subject to a much greater compressive load, leading to a factor of safety to stiffener crippling of 0.62.

**Fig. 18:**
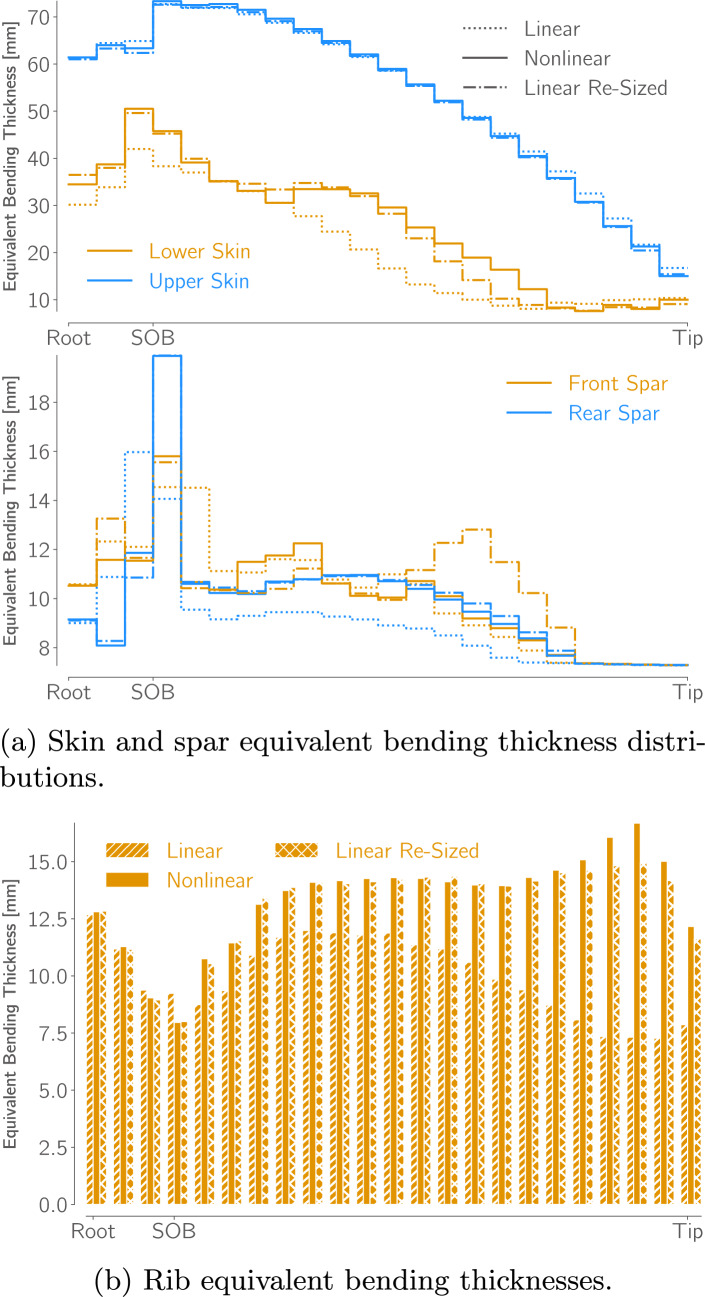
Sizing the structure using a nonlinear model results in lower skin, rib, and spar panels with higher bending stiffness due to Brazier effects

Figure 18 shows the distributions of effective bending thickness^27^ in the skin, spar, and rib panels of the linear and nonlinear optimized designs, and Table 8 shows a mass breakdown of each optimized wingbox. Because the nonlinear formulation captures Brazier effects and the loads they cause, the nonlinear optimized design’s ribs, rear spar, and lower skin have significantly higher bending stiffness. Despite the large difference in FOS, the spars in the nonlinear design are only 2.5% heavier than in the linear design, while the ribs had to be made 11.8% heavier.

**Table 8:** Wingbox mass breakdown for the linear and nonlinear optimized designs

Component	Linear	Nonlinear	% Change
Ribs	43.9	49.1	+11.8
Spars	69.1	70.8	+2.5
Upper skin	747.0	762.4	+2.1
Lower skin	668.5	698.0	+4.4
Total	1528.5	1580.3	+3.4

### Structural re-sizing

As mentioned in Sect. 2.3, the level of detail in the structural sizing in these optimizations is representative of conceptual or early preliminary design stages. More detailed sizing occurs later in the design process with more detailed models and more accurate internal loads. From an aerostructural design perspective, the key question is whether nonlinear effects on internal loads change structural mass enough to affect the drag-mass trade-off and optimum planform design. To investigate this, we re-optimize the structural sizing of the linear design using the nonlinear structural formulation, with the wing geometry fixed. We remove the wing loading and fuel volume constraints from this problem since the optimizer cannot change the wing geometry to satisfy them.

The lines labeled “linear re-sized” in Fig. 18 show the equivalent bending thickness distributions of the re-sized linear design. In the three areas where the linear and nonlinear designs’ panel bending stiffnesses differ most—the lower skin, rear spar, and ribs—the re-sized linear design is now much closer to the nonlinear design. However, as shown in Table 7, these changes in structural sizing have little effect on the overall aircraft performance. Specifically, choosing the “wrong” wing geometry based on a linear structural model before later re-sizing the structure with a nonlinear structural model costs only a 6.7kg (0.06%) increase in fuel burn and a 0.5kg (0.003%) increase in wingbox mass.^28^ This increase in fuel burn is equivalent to that caused by a 0.15 count increase in the aircraft’s drag coefficient which, according to Hoerner (1965), is less than the drag caused by two pitot tubes.^29^ This suggests that, while the structure eventually needs to be sized considering geometrically nonlinear effects, an accurate drag-mass trade-off and a good planform design can be achieved without considering them.

### Discussion

Compared to Calderon et al. (2019), our results predict a more negative impact of geometric nonlinearity on aircraft performance. They found that geometric nonlinearity leads to lighter and more efficient optimized designs, and increased the optimum aspect ratio by 5%. We found that the nonlinear design was heavier, similarly, if not less efficient, and that the optimum aspect ratio was only 1.6% higher. The primary reason for these contradictory findings is likely due to differences in structural modeling fidelity, among other variations in computational methods and aircraft specifications. They used a geometrically nonlinear beam, from which moments and shear forces were extracted and used to size the cross sections of the wingbox using analytical methods. This approach would not capture the Brazier effect and the resulting additional local stresses, which we found were the primary cause of the structural mass increase in the nonlinear design.

The optimization problem we solve here omits many constraints required for realistic wing design, particularly when allowing planform variation. These include landing gear packaging, trim, static stability, flutter stability, gust loads, control effectiveness, takeoff and landing performance, and buffet margin, among others. Aeroelastic constraints usually result in stiffer, heavier wings for a given planform. Combined with performance constraints limiting maximum aircraft weight (e.g., takeoff and landing distances), they reduce the optimum wing aspect ratio. Without these constraints, our optimizations result in designs with unrealistically high aspect ratios and large in-flight deflections. The purpose of these solutions is not to propose realistic design candidates, but to demonstrate the capabilities described in Sects. 1 and 2 and investigate geometric nonlinearity impacts in the static cruise and maneuver conditions we consider.

Despite such high in-flight deflections, and the fact that this optimization problem considered only static cruise and maneuver performance, the impact of geometrically nonlinear effects on the design was limited. Such effects in static cruise and maneuver conditions would likely have an even smaller impact on the design of a more realistic, and less flexible, wing. Additionally, the rib spacing in our optimized designs is high because we started with a low-aspect-ratio wing and stretched it into a high-aspect-ratio one. Because the rib crushing loads caused by Brazier effects are proportional to the rib spacing, the impacts of Brazier effects would be less severe in a wingbox with more realistic rib spacing.

While the strength of any given geometrically nonlinear effect reduces with the wing’s flexibility, this does not mean that the design of more realistic wings is unaffected by geometric nonlinearity. Including more flight points and aeroelastic constraints (such as those mentioned above), would only introduce more chances for geometric nonlinearity to affect the design in new ways, even at lower deflection levels. For example, in previous work (Gray et al. 2023) we found that geometrically nonlinear flutter phenomena can significantly affect the design of a wing exhibiting in-flight deflections as low as 5-10% semispan.

In that work, the geometrically nonlinear flutter constraint was computed using a lower fidelity representation of the wing, condensed from the kind of high-fidelity models presented here. Such mixed fidelity approaches [also proposed by Stodieck et al. (2018)] are necessary if the variety and number of aeroelastic constraints required for realistic aerostructural design are to be included in the kind of aerostructural optimization problems we presented here. A key challenge in such approaches is the implementation of a condensation process that can handle changes in the wing’s geometry during optimization, and that is differentiable with respect to all necessary design variables.

## Conclusions

Improving airframe technologies enable increasingly flexible aircraft wings, making it increasingly important to consider geometrically nonlinear effects of large in-flight deformation on aeroelastic performance. We develop tools for large-scale, gradient-based aerostructural optimization of high-aspect-ratio wings at a new fidelity, using RANS CFD aerodynamics and built-up geometrically nonlinear structural models. The geometrically nonlinear structural analysis capabilities we implement in the open-source FE library TACS include a novel geometrically nonlinear shell element formulation that uses quadratic rotation parameterization to capture moderate rotations in highly flexible aircraft wings. To solve the resulting nonlinear residual equations, we implement a nonlinear static structural solver using predictor-corrector continuation, adaptive linear convergence, and energy minimization for restarting analyses from previous solutions. We model stiffened composite panels typical of modern aerospace structures using a smeared stiffness approach. This approach includes stiffener cross sections and spacings in the optimization problem and incorporates criteria for multiple material and buckling failure modes. We couple TACS to CFD-based aerodynamics using the MELD transfer scheme, which correctly accounts for large deformations and rotations.

Using the multiphysics coupling library MPhys, we demonstrated what we believe to be the first simultaneous optimization of a wing’s aerodynamic shape and structural sizing using high-fidelity geometrically nonlinear models. The optimization problem aimed to minimize the fuel burn of a wing based on that of a Boeing 717 by varying 547 design variables, subject to 1277 constraints on structural failure, geometric properties, and structural sizing rules.

Despite the high aspect ratio (greater than 19) and in-flight deflection (greater than 35% semispan) of our optimized wing designs, introducing geometric nonlinearity to the optimization problem did not have a significant effect on the optimum wing’s planform, mass, aerodynamic efficiency, or passive load alleviation capability. The most critical way that geometric nonlinearity affected our results was in the distribution of internal forces in the wing structure. The nonlinear formulation captured the well-known rib crushing loads caused by the Brazier effect, but also captured additional local bending loads in the wing skins and spars, which reduced the safety margin of the structure designed using linear analysis from 1.5 to 0.62. Despite the greatly lower safety margins, the difference in overall structural mass between designs sized based on linear and nonlinear structural formulations was not enough to significantly alter the trade-off between drag and structural mass. Optimizing the wing’s shape using the linear structural formulation before re-sizing the structure (with a fixed geometry) using the nonlinear formulation only led to a 0.06% increase in fuel burn.

These results should not be extrapolated beyond this scenario. Further studies are required to determine whether our findings hold for other aircraft configurations and requirements, and when considering broader flight conditions and aeroelastic constraints. Nevertheless, our open-source tools enable researchers and aircraft manufacturers to pursue next-generation high-aspect-ratio wing designs by accurately predicting aeroelastic performance under extreme deformations.

## Figures and Tables

**Fig. 19:**
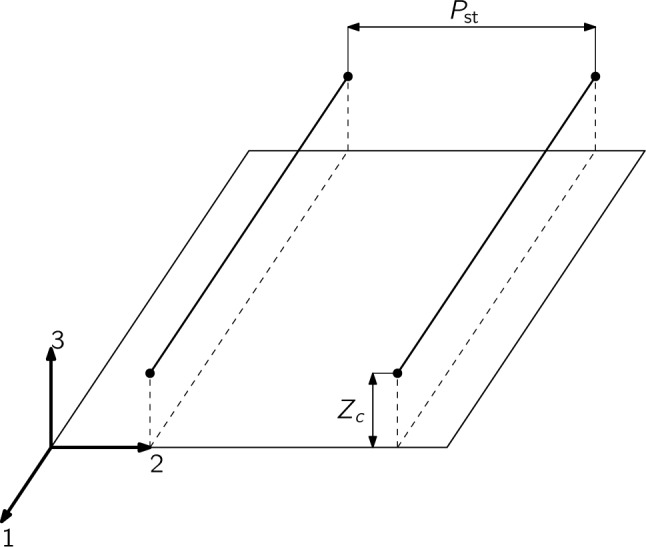
A stiffened shell panel with evenly spaced parallel stiffeners simplified to a 1D beam representation

## Data Availability

The data that support the findings of this study are available from the corresponding author upon request.

## A. Stiffened shell constitutive model

The constitutive model is based on the theory described by Nemeth (2011) and assumes the arrangement shown in Fig. 19, stiffeners are modeled as beams aligned with the local coordinate system of the shell, with a uniform pitch, $P_\text {st}$ and their centroids a distance $Z_c$ from the shell mid-plane.

Using first-order shear deformation theory, the strain field in the shell is parameterized by the mid-surface membrane strains, $\epsilon ^0_1, \epsilon ^0_2, \epsilon ^0_{12}$, mid-surface transverse shear strains, $\epsilon ^0_{23}, \epsilon ^0_{13}$ and the bending strains, $\kappa _1, \kappa _2, \kappa _{12}$, that describe the rate-of-change of the in-plane strains through the thickness of the shell

24 $$\begin{aligned} \begin{bmatrix}\epsilon _1(x_3) \\ \epsilon _2(x_3) \\ \epsilon _{12}(x_3)\end{bmatrix} = \begin{bmatrix}\epsilon ^0_1 \\ \epsilon ^0_2 \\ \epsilon ^0_{12}\end{bmatrix} + \begin{bmatrix}\kappa _1 \\ \kappa _2 \\ \kappa _{12}\end{bmatrix} x_3, \end{aligned}$$

where $x_3$ is the distance from the shell mid-plane. This results in the following stress–strain relations for the shell

25 $$\begin{aligned} \begin{bmatrix}N_1 \\ N_2 \\ N_{12}\end{bmatrix}= & \begin{bmatrix} A_{11} & A_{12} & A_{16} \\ A_{12} & A_{22} & A_{26} \\ A_{13} & A_{26} & A_{66} \\ \end{bmatrix} \begin{bmatrix}\epsilon ^0_1 \\ \epsilon ^0_2 \\ \epsilon ^0_{12}\end{bmatrix} \nonumber \\ & \quad + \begin{bmatrix} B_{11} & B_{12} & B_{16} \\ B_{12} & B_{22} & B_{26} \\ B_{13} & B_{26} & B_{66} \\ \end{bmatrix} \begin{bmatrix}\kappa _1 \\ \kappa _2 \\ \kappa _{12}\end{bmatrix} \end{aligned}$$

26 $$\begin{aligned} & \begin{bmatrix}M_1 \\ M_2 \\ M_{12}\end{bmatrix} = \begin{bmatrix} B_{11} & B_{12} & B_{16} \\ B_{12} & B_{22} & B_{26} \\ B_{13} & B_{26} & B_{66} \\ \end{bmatrix} \begin{bmatrix}\epsilon ^0_1 \\ \epsilon ^0_2 \\ \epsilon ^0_{12}\end{bmatrix} \nonumber \\ & \quad + \begin{bmatrix} D_{11} & D_{12} & D_{16} \\ D_{12} & D_{22} & D_{26} \\ D_{13} & D_{26} & D_{66} \\ \end{bmatrix} \begin{bmatrix}\kappa _1 \\ \kappa _2 \\ \kappa _{12}\end{bmatrix} \end{aligned}$$

27 $$\begin{aligned} \begin{bmatrix}Q_2 \\ Q_1\end{bmatrix} = \begin{bmatrix} A_{44} & A_{45} \\ A_{45} & A_{55}\end{bmatrix} \begin{bmatrix}\epsilon ^0_{23} \\ \epsilon ^0_{13}\end{bmatrix}, \end{aligned}$$

where, $N_i$, $Q_i$ and $M_i$ are the in-plane forces, transverse shear forces and bending moments per unit length. Or in block matrix form

28 $$\begin{aligned} \boldsymbol{\sigma _\text {sh}} = \boldsymbol{C_\text {sh}} \boldsymbol{\epsilon _\text {sh}} = \begin{bmatrix} \boldsymbol{A_\text {sh}} & \boldsymbol{B_\text {sh}} & \boldsymbol{0} \\ \boldsymbol{B_\text {sh}} & \boldsymbol{D_\text {sh}} & \boldsymbol{0} \\ \boldsymbol{0} & \boldsymbol{0} & \boldsymbol{A_{s,\text {sh}}} \end{bmatrix} \begin{bmatrix}\boldsymbol{\epsilon ^0} \\ \boldsymbol{\kappa } \\ \boldsymbol{\epsilon _s}\end{bmatrix}. \end{aligned}$$

Applying similar assumptions to the beam yields

29 $$\begin{aligned} \boldsymbol{\sigma }_\text {sh}= & \begin{bmatrix}F_1 \\ M_1 \\ M_2 \\ M_3 \\ V_{13} \\ V_{12}\end{bmatrix} = \boldsymbol{C}_\text {st} \boldsymbol{\epsilon }_\text {st} \nonumber \\= & \begin{bmatrix} C_{11} & C_{12} & C_{13} & C_{14} & C_{15} & C_{16} \\ C_{12} & C_{22} & C_{23} & C_{24} & C_{25} & C_{26} \\ C_{13} & C_{23} & C_{33} & C_{34} & C_{35} & C_{36} \\ C_{14} & C_{24} & C_{34} & C_{44} & C_{45} & C_{46} \\ C_{15} & C_{25} & C_{35} & C_{45} & C_{55} & C_{56} \\ C_{16} & C_{26} & C_{36} & C_{46} & C_{56} & C_{66} \\ \end{bmatrix} \begin{bmatrix} \epsilon _{11} \\ \chi _1 \\ \chi _2 \\ \chi _3 \\ \epsilon _{13} \\ \epsilon _{12} \end{bmatrix}, \end{aligned}$$

where *F*, *V*, *M* are the axial force, shear forces, and moments, $\epsilon _{11}, \epsilon _{12}, \epsilon _{13}$ are the axial and shear strains at the beam centroid, and $\chi _1, \chi _2, \chi _3$ are beam bending strains.

To compute the effective stiffness of the stiffened shell, the stiffener strains must be written in terms of the shell strains. This is done by extending the assumed strain variation through the thickness of the shell up to the centroid of the stiffener. The stiffener centroid strains can then be calculated from the shell mid-plane strains using a strain transformation matrix $\boldsymbol{T}_{\epsilon }$

30 $$\begin{aligned} \boldsymbol{\epsilon }_\text {st}= & \begin{bmatrix}\epsilon _{11} \\ \chi _1 \\ \chi _2 \\ \chi _3 \\ \epsilon _{13} \\ \epsilon _{12}\end{bmatrix} = \begin{bmatrix}\epsilon ^0_{1}+Z_c \kappa _1 \\ -\kappa _{12}/2 \\ \kappa _1 \\ 0 \\ \epsilon ^0_{13} \\ \left( \epsilon ^0_{12}+Z_c\kappa _{12}\right) /2\end{bmatrix} \nonumber \\= & \begin{bmatrix} 1 & 0 & 0 & Z_c & 0 & 0 & 0 & 0 \\ 0 & 0 & 0 & 0 & 0 & -1/2 & 0 & 0 \\ 0 & 0 & 0 & 1 & 0 & 0 & 0 & 0 \\ 0 & 0 & 0 & 0 & 0 & 0 & 0 & 0 \\ 0 & 0 & 0 & 0 & 0 & 0 & 0 & 1 \\ 0 & 0 & 1/2 & 0 & 0 & Z_c/2 & 0 & 0 \end{bmatrix} \begin{bmatrix} \epsilon ^0_1 \\ \epsilon ^0_2 \\ \epsilon ^0_{12} \\ \kappa _1 \\ \kappa _2 \\ \kappa _{12} \\ \epsilon ^0_{23} \\ \epsilon ^0_{13} \end{bmatrix} = \boldsymbol{T}_{\epsilon } \boldsymbol{\epsilon }_\text {sh}. \end{aligned}$$

The factor of 1/2 applied to the shear strains is to account for the fact that the stiffeners only stiffen the panel against shearing in the $x_1$ normal plane, and not the $x_2$ normal plane (Nemeth 2011).

The loads felt at the shell mid-plane due to the stresses in the stiffener can be calculated using the reverse transformation and accounting for the stiffener pitch to smear the stiffener’s stress contribution across the shell, resulting in the combined stress–strain relationship

31 $$\begin{aligned} \boldsymbol{\sigma } = \boldsymbol{\sigma }_\text {sh} + \frac{1}{P_\text {st}}\boldsymbol{T}^\text {T}_{\epsilon } \boldsymbol{\sigma }_\text {st} = \boldsymbol{\sigma }_\text {sh} + \frac{1}{P_\text {st}}\boldsymbol{T}^\text {T}_{\epsilon } \boldsymbol{C}_\text {st} \boldsymbol{\epsilon }_\text {st}. \end{aligned}$$

Combining Eq. (30) and (31) gives the combined stiffness matrix

32 $$\begin{aligned} \boldsymbol{C} = \boldsymbol{C}_\text {sh} + \frac{1}{P_\text {st}}\boldsymbol{T}^\text {T}_{\epsilon } \boldsymbol{C}_\text {st} \boldsymbol{T}_{\epsilon }. \end{aligned}$$

Equations (31) and (32) are valid for any method of computing the shell and stiffener stiffness matrices in Equations (28) and (29). In this implementation we model both the shell and stiffener as smeared laminates using a simplification of classical lamination theory. We define each laminate by its overall thickness, *t*, a discrete set of ply angles, $\theta _i$, and corresponding ply fractions, $f_i$ that define the fraction of the laminate thickness made up of each ply angle. We assume all plies have the same specially orthotropic properties defined by the lamina stiffness matrices $\boldsymbol{Q_\text {ply}},\boldsymbol{Q_{s, \text {ply}}}$

33 $$\begin{aligned} \begin{bmatrix}\sigma _{11} \\ \sigma _{22} \\ \tau _{12}\end{bmatrix} = \boldsymbol{Q_\text {ply}} \begin{bmatrix}\epsilon _{11} \\ \epsilon _{22} \\ \gamma _{12}\end{bmatrix}= & \begin{bmatrix} Q_{11} & Q_{12} & 0 \\ & Q_{22} & 0 \\ \text {sym} & & Q_{66} \end{bmatrix}\begin{bmatrix}\epsilon _{11} \\ \epsilon _{22} \\ \gamma _{12}\end{bmatrix} \nonumber \\= & \begin{bmatrix} \frac{E_1}{1-\nu _{12}\nu _{21}} & \frac{\nu _{12} E_2}{1 - \nu _{12}\nu _{21}} & 0 \\ & \frac{E_2}{1-\nu _{12}\nu _{21}} & 0 \\ \text {sym} & & G_{12} \end{bmatrix}\begin{bmatrix}\epsilon _{11} \\ \epsilon _{22} \\ \gamma _{12}\end{bmatrix} \end{aligned}$$

34 $$\begin{aligned} \begin{bmatrix}\tau _{23} \\ \tau _{13}\end{bmatrix}= & \boldsymbol{Q_{s, \text {ply}}} \begin{bmatrix}\gamma _{23} \\ \gamma _{13}\end{bmatrix} = \begin{bmatrix} Q_{44} & 0 \\ 0 & Q_{55} \end{bmatrix}\begin{bmatrix}\gamma _{23} \\ \gamma _{13}\end{bmatrix} \nonumber \\= & \begin{bmatrix} G_{23} & 0 \\ 0 & G_{13} \end{bmatrix}\begin{bmatrix}\gamma _{23} \\ \gamma _{13}\end{bmatrix}, \end{aligned}$$

where the 1,2,3 directions are the local material coordinate system of the ply. Assuming the plies of each angle are distributed uniformly through the laminate thickness, the laminate stiffness matrices are then a weighted sum of the stiffness matrices of each ply

35 $$\begin{aligned} \boldsymbol{Q_\text {lam}}&= \sum _{i=1}^N f_i \boldsymbol{\bar{Q}_\text {ply}}(\theta _i) \end{aligned}$$

36 $$\begin{aligned} \boldsymbol{Q_{s, \text {lam}}}&= \sum _{i=1}^N f_i \boldsymbol{\bar{Q}_{s, \text {ply}}}(\theta _i) \end{aligned}$$

37 $$\begin{aligned} \boldsymbol{A}&= t \boldsymbol{Q_\text {lam}} \end{aligned}$$

38 $$\begin{aligned} \boldsymbol{A_s}&= kt \boldsymbol{Q_{s, \text {lam}}} \end{aligned}$$

39 $$\begin{aligned} \boldsymbol{B}&=\boldsymbol{0} \end{aligned}$$

40 $$\begin{aligned} \boldsymbol{D}&= \frac{t^3}{12} \boldsymbol{Q_\text {lam}} , \end{aligned}$$

where $\boldsymbol{\bar{Q}}(\theta _i)$ is the lamina stiffness matrix rotated to the laminate coordinate system, and *k* is the shear correction factor that accounts for the non-uniform distribution of transverse shear through the laminate thickness. This approach is valid for thick laminates where the distribution of ply angles through the thickness is approximately uniform. Most importantly, the parameterization avoids the discontinuities inherent in discrete laminate stacking sequences (Macquart et al. 2016).

Assuming the stiffener is laid up symmetrically across the stiffener cross-section, the beam stiffness matrix contains no coupling terms, and its entries can be computed using effective moduli

41 $$\begin{aligned}&\boldsymbol{C}_\text {st} = \begin{bmatrix} A_\text {st}E_\text {st} & & & & & \\ & G_\text {st}J_\text {st} & & & & \\ & & E_\text {st}I_{22,\text {st}} & & & \\ & & & E_\text {st}I_{33,\text {st}} & & \\ & & & & kG_\text {st}A_\text {st} & \\ & & & & & kG_\text {st}A_\text {st} \\ \end{bmatrix} \end{aligned}$$

42 $$\begin{aligned}&E_\text {st} = Q_{11} - \frac{Q_{12}^2}{Q_{22}} \end{aligned}$$

43 $$\begin{aligned}&G_\text {st} = Q_{66}, \end{aligned}$$

where $A_\text {st}$ is the area, $I_{22,\text {st}}$ the second moment of area and $J_\text {st}$ the polar moment of inertia of the stiffener cross-section about its centroid.

Substituting Eqs. (30) and (41) into Eq. (32) gives the overall stiffness matrix shown in Eq. 10.

## Footnotes

1 The terms “aerostructural” and “aeroelastic” are often used interchangeably in relation to wing design optimization. We distinguish between aeroelastic optimization (structural design considering aeroelastic effects) and aerostructural optimization (simultaneous shape and structural design). A similar definition is used by Ma and Elham (2024).

2 https://github.com/smdogroup/tacs

3 These results can be reproduced using the code available at https://github.com/smdogroup/tacs/tree/master/examples/nonlinear_cantilever

4 We compare errors with respect to the Abaqus solution instead of the analytic solution to try and isolate the error caused by the rotation matrix approximation from the error caused by the finite element discretization.

5 These results can be reproduced using the code available at https://github.com/smdogroup/tacs/tree/master/examples/nonlinear_annulus

6 The mesh used in this case used $200 \times 200$ elements, as apposed to the $16 \times 16$ mesh shown in the referenced manuscript.

7 These results can be reproduced using the code available at https://github.com/smdogroup/tacs/tree/master/examples/nonlinear_hemisphere

8 also known as Uflyand–Mindlin plate theory for shells and Timoshenko theory for beams (Elishakoff 2019)

9 e.g., $SR=1/2$ means that the loads can be doubled before failure occurs.

10 We assume the biaxial strength term $F_{12}$ to be zero.

11 For the complete equations, see Table III of Stroud and Agranoff (1976) or Table 4 of Brooks et al. (2019).

12 https://github.com/OpenMDAO/MPhys

13 https://github.com/OpenMDAO/openmdao

14 https://github.com/mdolab/adflow

15 https://github.com/smdogroup/funtofem

16 https://github.com/mdolab/pygeo

17 While it is technically possible for a linear FE solver to fail to converge, even when using a complete LU decomposition, this typically only happens if the structure is improperly constrained.

18 https://github.com/mdolab/pyoptsparse

19 More details on the benchmark model and optimization problems can be found at https://mdobenchmarks.github.io/MDOAeroelasticBenchmark, accessed 2025-07-12.

20 These constraints apply between adjacent panels of the same type (skin-to-skin or spar-to-spar), not between different panel types (skin-to-spar).

21 We refer to the two optimization problems and their resulting designs as “linear” and “nonlinear” for brevity. In reality, both the coupled analyses and the optimization problem are nonlinear in both cases; only the structural formulation and the load and displacement transfer scheme are linear in the “linear” case.

22 The factor by which the nonlinear structural formulation increases the optimization time varies from 1.25 to 2.

23 A “major iteration” in SNOPT includes the computation of a new search direction and the performing of a line search, which involves multiple function evaluations.

24 The “feasibility” value reported by SNOPT is the maximum constraint violation divided by $\max \left( 1, ||\boldsymbol{x}||_{\infty }\right)$, where $\boldsymbol{x}$ is the design variable vector (Gill et al. 2007).

25 The “optimality” value reported by SNOPT is the $\ell _{\infty }$ norm of the Lagrangian gradient, accounting for variable bounds, again divided by  $\max \left( 1, ||\boldsymbol{x}||_{\infty }\right)$ (Gill et al. 2007).

26 The “Equivalent thickness" plotted on the wingbox panels is $t_\text {eq} = t_\text {panel}+A_\text {stiff}/P_\text {stiff}$, where $t_\text {panel}$ is the panel thickness, $A_\text {stiff}$ is the stiffener cross-sectional area, and $P_\text {stiff}$ is the stiffener pitch.

27 The equivalent bending thickness is the thickness of an unstiffened panel that would have the same bending stiffness as the stiffened panel in the stiffened direction.

28 Admittedly the linear re-sized design is heavier despite being slightly smaller, but the difference is still insignificant.

29 Based on Hoerner’s $C_dA$ value of 0.01 $\textrm{ft}^2$ for a single pitot tube.

## Declarations

### Replication of results

All the codes used in this work are open-source and available on GitHub. Table 9 lists the latest releases of the codes (at the time of writing) that can be used to reproduce our results.

**Table 9:** Software versions used in this work

Code	Repository	Version
TACS	https://github.com/smdogroup/tacs	v3.8.0
FUNtoFEM	https://github.com/smdogroup/funtofem	v0.3.9
ADflow	https://github.com/mdolab/adflow	v2.12.1
pyGeo	https://github.com/mdolab/pygeo	v1.15.0
pySpline	https://github.com/mdolab/pyspline	v1.5.2
IDwarp	https://github.com/mdolab/idwarp	v2.6.1
pyOptSparse	https://github.com/mdolab/pyoptsparse	v2.12.0
mdolab-baseclasses	https://github.com/mdolab/baseclasses	v1.8.1
MPhys	https://github.com/OpenMDAO/mphys	v2.0.0
OpenMDAO	https://github.com/OpenMDAO/OpenMDAO	v3.36.1
